# Proximity Labeling in *Caenorhabditis elegans* to Detect Neuronal Proteins During Memory Formation

**DOI:** 10.21769/BioProtoc.5821

**Published:** 2026-09-20

**Authors:** Aelon Rahmani, Yee Lian Chew

**Affiliations:** Flinders Health and Medical Research Institute, College of Medicine and Public Health, Flinders University, Adelaide, Australia

**Keywords:** *C. elegans*, Learning, Memory, Neuroscience, Protein proximity labeling, TurboID

## Abstract

Memory is a fundamental process, regulated by protein–protein interactions within neuronal proteome networks. Learning-dependent changes in specific brain regions important for memory have been detected by mass spectrometry, by comparing proteins from animals trained to learn with mock-trained controls. Detection through this method relies on relative protein abundance; brain dissection is readily available for macroscopic animals to spatially control protein identification by mass spectrometry. In the nematode *C. elegans*, however, such spatial control is limited due to its microscopic size, hindering its utilization in proteomics. A protocol to address this limitation would strengthen an already excellent model to study memory, given that many proteins for learning are evolutionarily conserved in the worm and single-cell expression is uniquely defined across all 302 neurons. We modified existing protocols to enable (i) proximity labeling detection of neuronal proteins in *C. elegans* and (ii) high-throughput enrichment of these proteins from >3,000 whole worm bodies simultaneously, to assess trained vs. mock-trained proteomes. This involved the biotin ligase enzyme TurboID, which promiscuously labels nearby proteins with its substrate biotin. Enzyme expression was transgenically restricted to the nervous system, and biotin supplementation was limited to the training (or mock training) period in a classical (gustatory) conditioning paradigm. Labeled proteins were enriched by pull-down using streptavidin, which has a high binding affinity to biotin, and then processed for mass spectrometry runs and qualitative data analysis. This protocol is uniquely advantageous in that it minimizes proteins present before a temporal window of interest (training/mock training), improving the detection of lowly abundant proteins from a specific tissue in the worm (neurons). We have demonstrated that the protocol can sufficiently detect novel learning regulators, thus providing a useful framework to interrogate proteomes in microscopic brains.

Key features

• This protocol assumes that users have fundamental knowledge about *C. elegans*.

• The entire workflow typically takes approximately three weeks, with many opportunities to pause and revisit the procedure.

• This protocol can be adapted for use in other animals and applied to a diverse array of temporally defined biological processes.

## Graphical overview


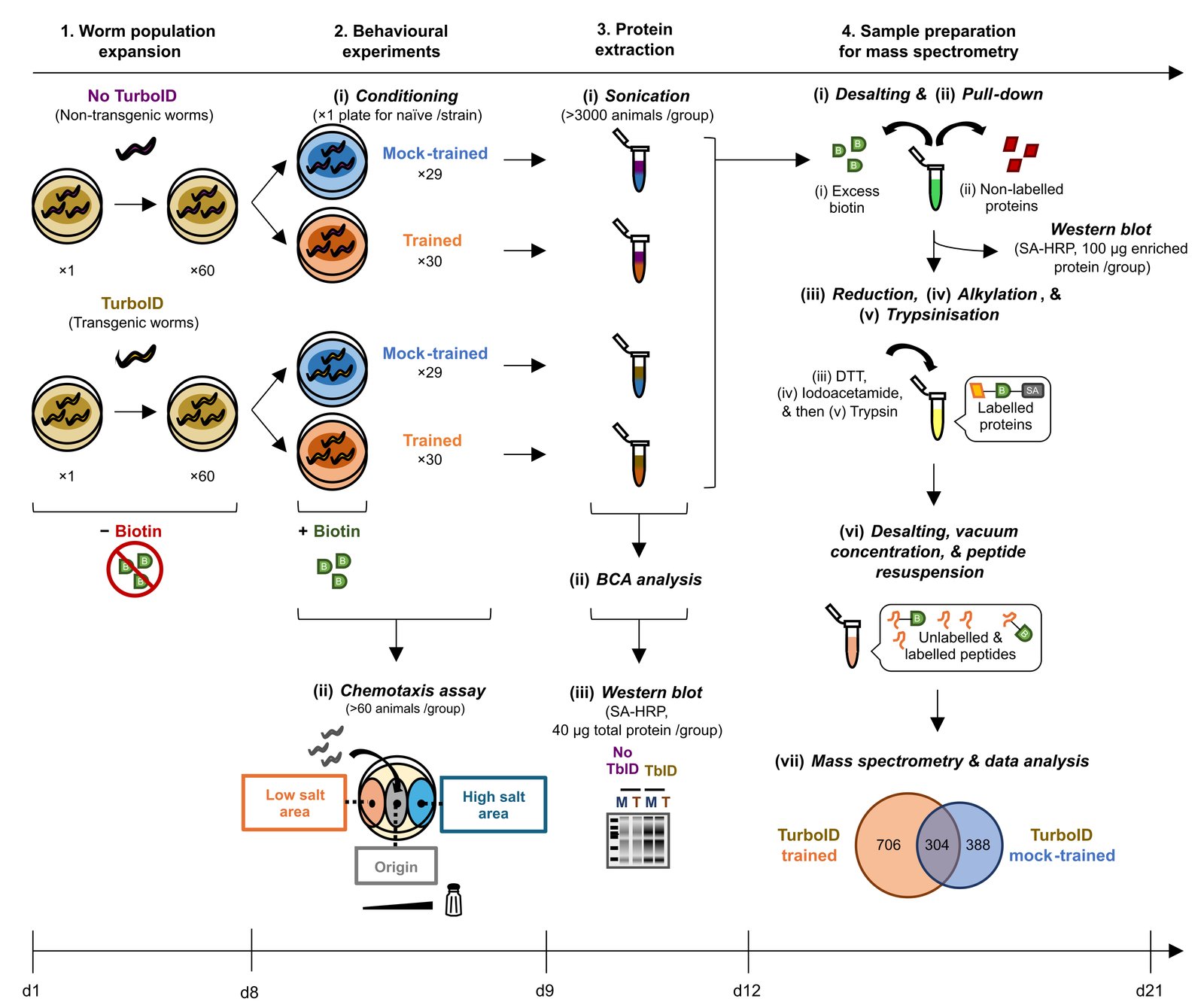


**Overview of the TurboID-based proximity labeling protocol in *C. elegans* neurons during classical conditioning**

## Background

The ability to learn is critical to ensure survival. This process is dependent on protein composition and its interactions within a proteome network. Generally, learned information is encoded in neurons as short-term memory (STM, from mass training) or long-term memory (LTM, from spaced training). Protein synthesis is crucial for LTM [1–5] and some forms of STM [1,2,6–8]. Animal studies have revealed the requirement for proteins in learning using the protein synthesis inhibitor cycloheximide [1–8], but this does not identify which proteins are important. Mass spectrometry has been used to compare brain regions from space-trained vs. untrained (or mock-trained) animals [9,10]. This approach identifies many proteins simultaneously. However, it does not directly discern which proteins regulate memory encoding vs. its storage or retrieval—distinct, temporally defined phases that are differently modulated [11–13]. To fully understand how memory is regulated relies on knowing how the network of interacting proteins functions in relevant cells (i.e., neurons) during specific phases of memory (e.g., memory encoding). This knowledge may inform treatment approaches for memory dysfunction in healthy aging and pathology.

We incorporated the existing proximity labeling tool TurboID to spatiotemporally dissect the neural proteome present during memory encoding in the nematode *Caenorhabditis elegans* [14], using mass spectrometry. *C. elegans* is an excellent system to study proteins important for learning and memory, given that many mechanisms for learning in the worm are conserved across species (reviewed in [15–17]), including protein synthesis [4–8]. However, their utility in proteomic experiments for high-throughput analysis is limited due to their microscopic size, with neurons constituting ~1% of their total volume [18]. This means that adequate detection by mass spectrometry requires neuronal proteins pooled from thousands of animals. TurboID employs a promiscuous biotin ligase to label proteins in proximity to the enzyme, contingent on the availability of its substrate, biotin [19]. This enables the selective enrichment of labeled proteins from large populations of animals for subsequent mass spectrometry analysis. TurboID was expressed pan-neuronally in *C. elegans* to achieve spatially restricted protein labeling. This leverages the worm’s amenability to genetic manipulation, well-characterized nervous system [20,21], and gene expression profiles known to the single neuron level [22–24]. To generate the transgenic strain used in this protocol, a plasmid encoding TurboID under an established pan-neuronal promoter was introduced into *C. elegans* through standard microinjection methods for germline transgenesis [25]. The resulting extrachromosomal array was inherited by progeny and enabled pan-neuronal expression of TurboID [26]. Biotin availability was restricted to the training period, during which animals were subjected to a classical conditioning paradigm [14]. Because both conditioning and TurboID labeling occur over several hours in *C. elegans* [25,27,28], this enables selective labeling of proteins during memory formation.

Many behavioral paradigms have been developed to model associative learning by classical conditioning in the worm, due to its ability to learn within hours (≤6 h) [4,6,8,28]. These experiments pair a novel stimulus (conditioned stimulus, CS) with a cue that naturally elicits a behavioral response (unconditioned stimulus, US). The paired exposure trains animals to associate both stimuli, leading them to exhibit the unconditioned response or UR toward the CS (conditioned response, CR). In this protocol, bacteria (their food source, an appetitive US) are paired with the absence of sodium chloride/salt (“no salt,” i.e., CS). Worms initially show high salt attraction (UR); after training, they lose this preference, displaying no bias toward either high or low salt (CR). This classical conditioning protocol relies on the animals’ innate attraction to high salt concentrations (~50 mM) experienced alongside abundant food during their cultivation [29]. It is an example of salt associative learning [28] that is compatible with proximity labeling due to its temporal requirements [14].

The benefit of using this protocol is that it can reveal novel proteins that likely would not have been assessed following other screening methods. We demonstrate this in our recent publication [14]. To summarize, this high-throughput method was able to identify >700 proteins from neurons that were unique to trained animals (no salt + food) vs. mock-trained animals (salt + food). This learning proteome (with >87% neuronal proteins) included (1) key regulatory pathways for memory, (2) many proteins not yet studied in the context of learning, and (3) notable proteome representation from (i) neuron classes that modulate gustatory learning and (ii) those that have never before been implicated in learning. We also confirmed several proteins from this proteome data as novel learning regulators by assessing the learning capacity of single gene mutants [14]. TurboID can be adapted to target any biological context with defined spatial and temporal resolution. This publication complements our recent work in that it explains step-by-step how we performed proximity labeling in the worm. We provide this protocol to researchers as a resource to enable more precise proteomic studies in diverse experimental systems.

## Materials and reagents

**Biological materials**

1. *E. coli* OP50 (*Caenorhabditis* Genetics Center) (commercially available, RRID: WBStrain00041969)

2. *E. coli* MG1655 *bioB::kan* (Prof. John E. Cronan, School of Molecular & Cellular Biology, University of Illinois Urbana-Champaign) (commercially available via *Caenorhabditis* Genetics Center, RRID: WBStrain00062723)

3. *C. elegans* wild-type line Bristol N2 (*Caenorhabditis* Genetics Center) (commercially available, RRID: WBStrain00000001)

4. *C. elegans* transgenic *pepEx025[Prab-3::V5::TurboID::NES; Punc-122::RFP]* line YLC207 (requestable via A/Prof Yee Lian Chew, College of Medicine and Public Health, Flinders University) (not commercially available, no RRID)

**Reagents**

Note that these reagents are available from many distributors beyond those listed below. We have not tested this extensively, but buffer components (e.g., acids, bases, salts) can likely be sourced from alternative suppliers without substantially affecting experimental outcomes. In contrast, specialized reagents used for protein enrichment, digestion, and mass spectrometry sample preparation may be more sensitive to differences in formulation, quality, or purity. We recommend that users perform pilot experiments when they introduce alternatively supplied reagents, before larger-scale work.

1. Acetic acid, ≥99.7% purity (Chem-Supply, catalog number: AA009)

2. Acetonitrile, ≥99.9% purity (Chem-Supply, catalog number: LC1005)

3. Agar powder (Chem-Supply, catalog number: AL027)

4. Ammonium bicarbonate powder, ≥99.0% purity (NH_4_HCO_3_) (Merck, catalog number: A6141); prepare 50 mM NH_4_HCO_3_ in ultrapure water

5. β-glycerol phosphate disodium salt, ≥98.0% purity (Merck, catalog number: 50020); prepare 100 mM β-glycerol phosphate in ultrapure water

6. β-mercaptoethanol, 99.0% purity (Merck, catalog number: M3148)

7. Bacto^TM^ Tryptone powder (Thermo Fisher Scientific, catalog number: 211705)

8. Biotin powder, ≥99.0% purity (Merck, catalog number: B4639)

9. Bromophenol blue (Bio-Rad, catalog number: 1610404)

10. Calcium chloride powder (CaCl_2_) (Chem-Supply, catalog number: CA033); prepare 1 M CaCl_2_ in ultrapure water and autoclave

11. Cholesterol powder (Astral Scientific, catalog number: BIOCD0122); prepare 5 mg/mL cholesterol in 100% (v/v) molecular-grade ethanol

12. cOmplete^TM^ Protease Inhibitor Cocktail (Merck, catalog number: 11836153001); prepare 50× cOmplete^TM^ Protease Inhibitor Cocktail in ultrapure water

13. Dithiothreitol powder, 97.0% purity (DTT) (Merck, catalog number: 10197777001); prepare 1 M DTT in ultrapure water

14. Ethylenediaminetetraacetic acid powder, ≥99.0% purity (EDTA) (Chem-Supply, catalog number: EA023); prepare 0.5 M EDTA in ultrapure water

15. Ethanol, 80% (v/v) (Chem-Supply, catalog number: EL156)

16. Formic acid, 100% (v/v) (Chem-Supply, catalog number: AC10760100)

17. Glycerol, ≥99.5% purity (Chem-Supply, catalog number: GA010); prepare 50% (v/v) glycerol in nuclease-free water

18. Heptafluorobutyric acid, ≥99.5% purity (HFBA) (Merck, catalog number: 52411)

19. Hydrochloric acid, 37.0% (v/v) (HCl) (Merck, catalog number: 320331)

20. Iodoacetamide powder, ≥99.0% purity (Merck, catalog number: I6125); prepare 0.5 M iodoacetamide in ultrapure water

21. Potassium phosphate dibasic powder (K_2_HPO_4_) (Astral Scientific, catalog number: BIOPB0447)

22. Kanamycin powder, ≥750 IU/mg purity (Kan) (Life Technologies, catalog number: GA0531); prepare 50 mg/mL kanamycin in ultrapure water

23. Potassium chloride powder, ≥99.0% purity (KCl) (Chem-Supply, catalog number: PL054); prepare 1 M KCl in ultrapure water

24. Potassium phosphate monophosphate powder (KH_2_PO_4_) (Astral Scientific, catalog number: BIOPB0445)

25. Potassium hydroxide powder (KOH) (Ajax Chemicals, catalog number: 405); prepare 1 M KOH in ultrapure water

26. Magnesium sulfate powder (MgSO_4_) (Astral Scientific, catalog number: MA048); prepare 1 M MgSO_4_ autoclaved in ultrapure water

27. Methanol, ≥99.0% purity (Merck, catalog number: 34860); prepare 50% (v/v) methanol in ultrapure water

28. Molecular-grade ethanol, ≥99.5% purity (Chem-Supply, catalog number: EA043)

29. Nuclease-free water (Merck, catalog number: W3500)

30. Nonidet-P40, 100% (v/v); no longer in production, so instead use IGEPAL^®^ CA-630 (Merck, catalog number: I8896)

31. Pierce^TM^ BCA^®^ Protein Assay kit reagents (Thermo Fisher Scientific, catalog number: 23227)

32. Phosphate-buffered saline (PBS), pH 7.4 (Thermo Fisher Scientific, catalog number: 10010023)

33. ProteaseMAX surfactant powder (Promega, catalog number: V2072); prepare 1% (w/v) ProteaseMAX in 50 mM NH_4_HCO_3_

34. Sodium azide powder, ≥99.5% purity (NaN_3_) (Merck, catalog number: S2002); prepare 0.1 M NaN_3_ in ultrapure water

35. Sodium carbonate powder, ≥99.9% purity (Na_2_CO_3_) (Merck, catalog number: 1063920500); prepare 0.1 M Na_2_CO_3_ in ultrapure water

36. Sodium chloride powder, ≥99.0% purity (NaCl) (Chem-Supply, catalog number: SA046); prepare 2 M NaCl autoclaved in ultrapure water

37. Sodium deoxycholate powder, ≥97.0% purity (Merck, catalog number: D6750); prepare 6% (w/v) sodium deoxycholate in ultrapure water

38. Sodium dodecyl sulfate powder (SDS) (Merck, catalog number: 428015); prepare 10% (w/v) SDS in ultrapure water

39. Sodium fluoride, ≥99.0% purity (Merck, catalog number: 201154); prepare 1 M sodium fluoride in ultrapure water

40. Sodium orthovanadate powder, ≥99.98% purity (Merck, catalog number: 450243); prepare 200 mM sodium orthovanadate in ultrapure water

41. Sodium pyrophosphate decahydrate powder, ≥99.0% purity (Thermo Fisher Scientific, catalog number: ACR205970250); prepare 100 mM sodium pyrophosphate in ultrapure water

42. Streptavidin-coated magnetic beads (New England Biolabs, catalog number: S1420S)

43. Trifluoroacetic acid, ≥99.0% purity (TFA) (Merck, catalog number: T6508); prepare 0.1% (w/v) TFA and 10% (w/v) TFA in ultrapure water

44. Tris(hydroxymethyl)methylamine powder, ≥99.8% purity (Tris) (Chem-Supply, catalog number: TA034); prepare 1 M Tris-Cl pH 6.8, pH 7.4, and pH 8.0 in ultrapure water (pH-adjusted with HCl)

45. Trypsin powder and resuspension buffer (Promega, catalog number: V5111); prepare 0.2 μg/μL trypsin in trypsin resuspension buffer

46. Tween-20, 100% (v/v) (Merck, catalog number: P1379)

47. Urea powder, ≥99.0% purity (Merck, catalog number: U5128)

48. Yeast extract powder (Glentham Life Sciences, catalog number: GE4420)

**Solutions**

1. Biotin, 0.1 M (see Recipes)

2. Cartridge elution (CE) solution (see Recipes)

3. Cartridge washing (CW) solution (see Recipes)

4. Digestion solution (see Recipes)

5. K_3_PO_4_, 1 M, pH 6.0 (see Recipes)

6. Luria broth (LB), standard version (see Recipes)

7. LB agar (see Recipes)

8. Modified LB (see Recipes)

9. Mock-training agar (MT agar) (see Recipes)

10. Mass spectrometry resuspension buffer (MSRB) (see Recipes)

11. Nematode growth medium agar (NGM agar) (see Recipes)

12. No salt buffer (NSB) (see Recipes)

13. Protein reducing (PR) solution (see Recipes)

14. Radioimmunoprecipitation assay solution (RIPA) (see Recipes)

15. Salt-deficient agar (SD agar) (see Recipes)

16. Salt gradient agar (SG agar) (see Recipes)

17. Sample buffer, 5× (see Recipes)

18. Tris-buffered saline with Tween-20 (TBST) (see Recipes)

19. Urea, 5 M (see Recipes)

20. Urea-containing RIPA with high SDS (u-RIPA) (see Recipes)

21. Worm washing buffer (WWB) (see Recipes)

**Recipes**

**1. Biotin, 0.1 M**

**Table:** 

Reagent	Final concentration	Quantity or volume
Biotin powder	100 mM	0.0244 g
KOH, 1 M	250 mM	250 μL
Total (prepared in NSB)	n/a	1.0 mL

**2. CE solution** [30]

**Table:** 

Reagent	Final concentration	Quantity or volume
Acetonitrile, 100% (v/v)	80% (v/v)	40 mL
Acetic acid, 100% (v/v)	0.5% (v/v)	250 μL
Total (prepared in ultrapure water)	n/a	50 mL

**3. CW solution** [30]

**Table:** 

Reagent	Final concentration	Quantity or volume
Acetonitrile, 100% (v/v)	50% (v/v)	25 mL
Acetic acid, 100% (v/v)	0.5% (v/v)	250 μL
Total (prepared in ultrapure water)	n/a	50 mL

**4. Digestion solution** [30]

**Table:** 

Reagent	Final concentration	Quantity or volume
ProteaseMAX, 1% (w/v)	~0.02% (w/v)	2.5 μL
Trypsin, 0.2 μg/μL	~0.01 μg/μL	10 μL
Total (prepared in 50 mM of NH_4_HCO_3_)	n/a	162.5 μL

**5. K_3_PO_4_ 1 M, pH 6.0** [31]

**Table:** 

Reagent	Final concentration	Quantity or volume
KH_2_PO_4_ powder	796 mM	108.3 g
K_2_HPO_4_ powder	204 mM	35.6 g
Total (powders autoclaved in ultrapure water)	n/a	1,000 mL

**6. LB, standard version**

**Table:** 

Reagent	Final concentration	Quantity or volume
Bacto^TM^ Tryptone powder	1.0% (w/v)	2.0 g
NaCl powder	1.0% (w/v)	2.0 g
Yeast extract powder	0.5% (w/v)	1.0 g
Total (powders autoclaved in ultrapure water)	n/a	200 mL

**7. LB agar**

**Table:** 

Reagent	Final concentration	Quantity or volume
Agar powder	1.6% (w/v)	1.6 g
Bacto^TM^ Tryptone powder	1.0% (w/v)	1.0 g
NaCl powder	1.0% (w/v)	1.0 g
Yeast extract powder	0.5% (w/v)	0.5 g
Total (powders autoclaved in ultrapure water)	n/a	100 mL

**8. Modified LB**

**Table:** 

Reagent	Final concentration	Quantity or volume
Bacto^TM^ Tryptone powder	1.0% (w/v)	1.0 g
Yeast extract powder	0.5% (w/v)	0.5 g
NaCl, 2 M	25 mM	1.25 mL
K_3_PO_4_, 1 M	5 mM	625 μL
CaCl_2_, 1 M	1 mM	100 μL
MgSO_4_, 1 M	1 mM	100 μL
Kanamycin, 50 mg/mL	0.05 mg/mL	100 μL
Total (powders autoclaved in ultrapure water, before adding solutions)	n/a	100 mL

**9. MT agar** [28]

**Table:** 

Reagent	Final concentration	Quantity or volume
Agar powder	2% (w/v)	16.0 g
NaCl, 2 M	100 mM	40 mL
K_3_PO_4_, 1 M	5 mM	4.0 mL
CaCl_2_, 1 M	1 mM	0.8 mL
MgSO_4_, 1 M	1 mM	0.8 mL
Total (powder autoclaved in ultrapure water, before adding solutions)	n/a	800 mL

**10. MSRB** [30]

**Table:** 

Reagent	Final concentration	Quantity or volume
Formic acid, 100% (v/v)	1.0% (v/v)	10 μL
HFBA, 100% (v/v)	0.2% (v/v)	2 μL
Total (prepared in autoclaved ultrapure water)	n/a	1 mL

**11. NGM agar** [31]

**Table:** 

Reagent	Final concentration	Quantity or volume
Agar powder	1.7% (w/v)	17.0 g
Bacto^TM^ Tryptone powder	0.25% (w/v)	2.5 g
NaCl powder	50 mM	3.0 g
K_3_PO_4_, 1 M	25 mM	25 mL
CaCl_2_, 1 M	1 mM	1 mL
MgSO_4_, 1 M	1 mM	1 mL
Cholesterol, 5 mg/mL	0.005 mg/mL	1 mL
Total (powders autoclaved in ultrapure water, before adding solutions)	n/a	1,000 mL

**12. NSB** [32]

**Table:** 

Reagent	Final concentration	Quantity or volume
K_3_PO_4_, 1 M	5 mM	1 mL
CaCl_2_, 1 M	1 mM	200 μL
MgSO_4_, 1 M	1 mM	200 μL
Total (prepared in autoclaved ultrapure water)	n/a	200 mL

**13. PR solution** [30]

**Table:** 

Reagent	Final concentration	Quantity or volume
ProteaseMAX, 1% (w/v)	0.1% (w/v)	2.0 μL
NH_4_HCO_3_, 50 mM	7 mM	28 μL
Urea, 5 M	2 M	80 μL
DTT, 1 M	5 mM	1.1 μL
Total (prepared in ultrapure water)	n/a	200 μL

**14. RIPA, pH 8.0** [30]

**Table:** 

Reagent	Final concentration	Quantity or volume
Nonidet-P40, 100% (v/v) [or IGEPAL^®^ CA-630, 100% (v/v)]	1.0% (v/v)	300 μL
SDS, 10% (w/v)	0.1% (w/v)	300 μL
Sodium deoxycholate, 6% (w/v)	0.5% (w/v)	2.50 mL
NaCl, 2 M	150 mM	2.25 mL
Tris-Cl, 1 M (pH 8.0)	50 mM	1.50 mL
Sodium fluoride, 1 M	10 mM	300 μL
EDTA, 0.5 M	5 mM	300 μL
Sodium orthovanadate, 200 mM	2 mM	300 μL
β-glycerol phosphate, 100 mM	1 mM	300 μL
Sodium pyrophosphate, 100 mM	1 mM	300 μL
cOmplete^TM^ Protease Inhibitor Cocktail, 50×	1×	600 μL
Total (prepared in ultrapure water)	n/a	30 mL

**15. SD agar** [for chemotaxis (CTX) assay and training plates] [32]

**Table:** 

Reagent	Final concentration	Quantity or volume
Agar powder	2% (w/v)	20.0 g
K_3_PO_4_, 1 M	5 mM	5.0 mL
CaCl_2_, 1 M	1 mM	1.0 mL
MgSO_4_, 1 M	1 mM	1.0 mL
Total (powder autoclaved in ultrapure water, before adding solutions)	n/a	1000 mL

**16. SG agar** [33]

**Table:** 

Reagent	Final concentration	Quantity or volume
Agar powder	2% (w/v)	1.0 g
NaCl, 2 M	200 mM	5 mL
K_3_PO_4_, 1 M	5 mM	250 μL
CaCl_2_, 1 M	1 mM	50 μL
MgSO_4_, 1 M	1 mM	50 μL
Total (powder autoclaved in ultrapure water, before adding solutions)	n/a	50 mL

**17. Sample buffer, 5×**

**Table:** 

Reagent	Final concentration	Quantity or volume
Glycerol	50% (v/v)	500 μL
β-mercaptoethanol	2.5% (v/v)	25 μL
SDS powder	10 (w/v)	0.1 g
Bromophenol blue	0.25% (w/v)	0.0025 g
Tris-Cl (pH 6.8), 1 M	250 mM	250 μL
Total (prepared in ultrapure water)	n/a	1 mL

Incubate at 50–65 °C if powders do not dissolve. Prepare 1× sample buffer in RIPA.

**18. TBST, pH 7.4** [30]

**Table:** 

Reagent	Final concentration	Quantity or volume
Tween-20, 100% (v/v)	0.1% (v/v)	500 μL
NaCl, 2 M	150 mM	37.5 mL
Tris-Cl (pH 7.4), 1 M	10 mM	5 mL
Total (powders autoclaved in ultrapure water)	n/a	500 mL

**19. Urea, 5 M (pH 8.0)** [26]

**Table:** 

Reagent	Final concentration	Quantity or volume
Urea powder	5 M	0.6 g
Tris-Cl (pH 8.0), 1 M	6.25 mM	12.5 μL
Total (prepared in ultrapure water)	n/a	2 mL

Initially, add 1.25 mL of ultrapure water to urea powder with Tris-Cl in a 15 mL centrifuge tube and then shake the tube vigorously. The solution volume will increase to ~2 mL, since urea is hygroscopic.

**20. u-RIPA, pH 8.0** [25,30]

**Table:** 

Reagent	Final concentration	Quantity or volume
Nonidet-P40, 100% (v/v) [or IGEPAL^®^ CA-630, 100% (v/v)]	1.0% (v/v)	100 μL
SDS, 10% (w/v)	1.0% (w/v)	1 mL
Sodium deoxycholate, 6% (w/v)	0.5% (w/v)	833 μL
Urea, 5 M	2 M	4 mL
NaCl, 2 M	150 mM	750 μL
Tris-Cl 1 M (pH 8.0)	50 mM	500 μL
Sodium fluoride, 1 M	10 mM	100 μL
EDTA, 0.5 M	5 mM	100 μL
Sodium orthovanadate, 200 mM	2 mM	100 μL
β-glycerol phosphate, 100 mM	1 mM	100 μL
Sodium pyrophosphate, 100 mM	1 mM	100 μL
cOmplete^TM^ Protease Inhibitor Cocktail, 50×	1×	200 μL
Total (prepared in ultrapure water)	n/a	10 mL

**21. WWB**

**Table:** 

Reagent	Final concentration	Quantity or volume
NaCl, 2 M	50 mM	18.75 mL
K_3_PO_4_, 1 M	5 mM	3.75 mL
CaCl_2_, 1 M	1 mM	0.75 mL
MgSO_4_, 1 M	1 mM	0.75 mL
Total (prepared in autoclaved ultrapure water)	n/a	750 mL

**Laboratory supplies**

1. Airtight storage box, plastic, 7,500 mL (Coles, catalog number: 3794534)

2. Beaker, 50 mL (Kartell Labware, catalog number: 1822)

3. Bottle, clear borosilicate glass, 2,000 mL (Adelab Scientific, catalog number: LABCO355.012.902)

4. Bottle, clear borosilicate glass, 1,000 mL (Rowe Scientific, catalog number: GB8055)

5. Bottle, clear borosilicate glass, 500 mL (Rowe Scientific, catalog number: GB8005)

6. Bottle, clear borosilicate glass, 250 mL (Adelab Scientific, catalog number: LABCO355.012.250)

7. Bottle, clear borosilicate glass, 100 mL (Rowe Scientific, catalog number: GB7910)

8. Centrifuge tubes, 50 mL with V-bottom (Adelab Scientific, catalog number: ACT-50-B-S)

9. Centrifuge tubes, 15 mL with V-bottom (Adelab Scientific, catalog number: AXYSCT-15ML-25-S)

10. Centrifuge tubes, 2.0 mL with square bottom (Adelab Scientific, catalog number: AXYMCT-200-C)

11. Centrifuge tubes, 1.5 mL with V-bottom (Rowe Scientific, catalog number: PT0159)

12. Desalting spin columns, 7 kDa molecular weight cutoff (Thermo Fisher Scientific, catalog number: 89883)

13. Filtered low-retention pipette tips, 200 μL (Adelab Scientific, catalog number: AXYTF-200-L-R-S)

14. Glass serological pipette tips, 5 mL (Adelab Scientific, catalog number: TECH-LW3975-01)

15. Kimwipes (Adelab Scientific, catalog number: KIM34120L)

16. LC–MS/MS-compatible vials and their lids (Rowe Scientific, catalog numbers: VV0095 and VC0390, respectively)

17. Marker, 1.0 mm (Officeworks, catalog number: SA20083066)

18. Markers, 0.3 mm (Officeworks, catalog number: SH37175PP)

19. Petri dishes, 90 mm (Rowe Scientific, catalog number: PD0064)

20. Petri dishes, 60 mm (Rowe Scientific, catalog number: PP1194)

21. Razors (Rowe Scientific, catalog number: HS0278)

22. Ruler (Officeworks, catalog number: KJJSR3021)

23. Serological pipette tips, 25 mL (Rowe Scientific, catalog number: PP0190)

24. Serological pipette tips, 10 mL (Rowe Scientific, catalog number: PP0189)

25. Serological pipette tips, 1 mL (Interpath Services, catalog number: 604181)

26. Sheet protectors, A4 (Officeworks, catalog number: KESP10)

27. Syringes, 3 mL (Sigma-Aldrich, catalog number: Z116858)

28. Tape, 18 × 50 mm (Officeworks, catalog number: DK240350)

29. tC18 cartridges (Waters, catalog number: WAT036810)

30. Unfiltered pipette tips, 1,000 μL (Rowe Scientific, catalog number: DL3959)

31. Unfiltered pipette tips, 200 μL (Rowe Scientific, catalog number: PT0242)

32. Unfiltered pipette tips, 10 μL (Rowe Scientific, catalog number: DT0063)

## Equipment

1. 96-well microplate reader (Molecular Devices, model: SpectraMax iD5 Reader)

2. Centrifuge, mini (Rowe Scientific, model: IC0039)

3. Centrifuge, refrigerated (Eppendorf, model: 5425R)

4. Centrifuge tube rack (Adelab Scientific, model: LABCO650.100.210)

5. Click counter (Officeworks, model: JBTALLYCNT)

6. Direct sonication system and compatible sonicator probe (Qsonica, models: Q125 and CL-18, respectively)

7. Freezer, 700 L (Rowe, model: Bonvue)

8. Freezer, 440 L (Eppendorf, model: F440h, CryoCube)

9. Incubator, 520 L (Rowe, model: II0184)

10. Incubator, 300 L (Rowe, model: II0185)

11. Intelli-Mixer^TM^ and its 1.5–2.0 mL centrifuge tube rack (ELMI, model: RM-2M and 13 mm test tube rack)

12. Magnetic tube rack (New England Biolabs, catalog number: S1506S)

13. Manual pipette, 100–1,000 μL (Interpath Services, model: 8251000, Socorex)

14. Manual pipette, 20–200 μL (Interpath Services, model: 8250200, Socorex)

15. Manual pipette, 2–20 μL (Interpath Services, model: 8250020, Socorex)

16. Manual pipette, 0.5–10 μL (Interpath Services, model: 8250010, Socorex)

17. Orbitrap Exploris (Thermo Fisher Scientific, model: BRE725539)

18. Peristaltic pump and compatible footswitch (Rowe Scientific, model: IP0946 and IL0071)

19. Q-Exactive Orbitrap (Thermo Fisher Scientific, model: IQLAAEGAAPFALGMBDK)

20. Refrigerator, 570 L (Rowe, model: HR600, Nuline)

21. Scissors (Officeworks, catalog number: SMCG4)

22. Serological pipette controller (Interpath Services, model: 847070, Greiner Bio-One Maxipette)

23. Stainless steel laboratory spoons (Sigma-Aldrich, model: Z648299, equivalent suggested since product information is not available for spoons used in lab)

24. Stereomicroscope to view N2 *C. elegans* (Nikon, model: SM2715)

25. Stereomicroscope and light engine to view transgenic (YLC207) *C. elegans* (Nikon, models: SMZ1270 and Sola 80-10368)

26. Thermomixer (Eppendorf, model: F1.5)

27. Timer (Thermo Fisher Scientific, model: 1464917)

28. Tweezers, ~10 cm long, stainless steel with pointed tips (Sigma-Aldrich, model: Z648299, equivalent suggested since product information is not available for tweezers used in lab)

29. Vacuum concentrator (John Morris Scientific, model: Martin Christ RVC 2-25 CD plus)

30. Vortex shaker (Abdos Life Sciences, model: Swirlex)

31. Water bath (12 L) with its compatible lid (Rowe Scientific, model: IW0129 and IW0131)

## Software and datasets

1. Anaconda (Anaconda Software Distribution, version 2023.3): https://www.anaconda.com/download [RRID: SCR_025572]

2. Batch Entrez^®^ (https://www.ncbi.nlm.nih.gov/sites/batchentrez, accessed 2021-2023, free-to-use) [RRID: SCR_016634]

3. Excel (Microsoft Corporation, version 2603): https://www.microsoft.com/en-au/microsoft-365/excel (an equivalent software can be used to read and edit spreadsheets) [RRID:SCR_016137]

4. GraphPad Prism (GraphPad, version 8.0.2): https://www.graphpad.com/features (newer version commercially available; an equivalent statistics software can be used to assess data from chemotaxis assays) [RRID:SCR_002798]

5. MASCOT search engine (Matrix Science, version 2.8): https://www.matrixscience.com/ (newer version commercially available) [RRID:SCR_014322]

6. Python (The Python Software Foundation, version 3.9): https://www.python.org/downloads/ (free-to-use) [RRID:SCR_008394]

*Note: You will need to install the following packages to run the custom Python code from this protocol: BioPython (version 1.78) [34], bs4, csv, datetime, html5lib, os, pandas (version 1.5.3), pathlib, and shutil (free-to-use). This can be done by entering the following in a terminal using the command prompt .exe file available on all computers: pip install (package name), but replace ‘(package name)’ with one of the package names listed above.*

7. STRING (https://string-db.org/, accessed 2021–2023, version 12, free-to-use) [RRID:SCR_005223]

8. Venn diagram tool (https://bioinformatics.psb.ugent.be/webtools/Venn/, accessed 2021–2023, free-to-use)

9. VS code (Visual Studio Code, version 1.120.0): https://code.visualstudio.com/download (free-to-use) [RRID:SCR_026031]

10. Windows Clock (Microsoft Corporation, version 2026): https://apps.microsoft.com/detail/9wzdncrfj3pr?hl=en-US&gl=IL (free-to-use)

11. The custom Python code files have been deposited to GitHub: https://github.com/ChewWormLab/Chew-Worm-Lab-Post-Mass-Spectrometry-Peptide-processing (free-to-use, given the related research paper is appropriately referenced in future papers using the code)

## Procedure

**A. Preparation of bacteria**

*Note: Bacteria that are purchased or requested are typically in the form of colonies on LB agar plates. These will need to be cultured to grow more bacteria. LB agar plates are prepared in 90 mm Petri dishes (~25 mL of agar per plate).*

**Figure 1.**
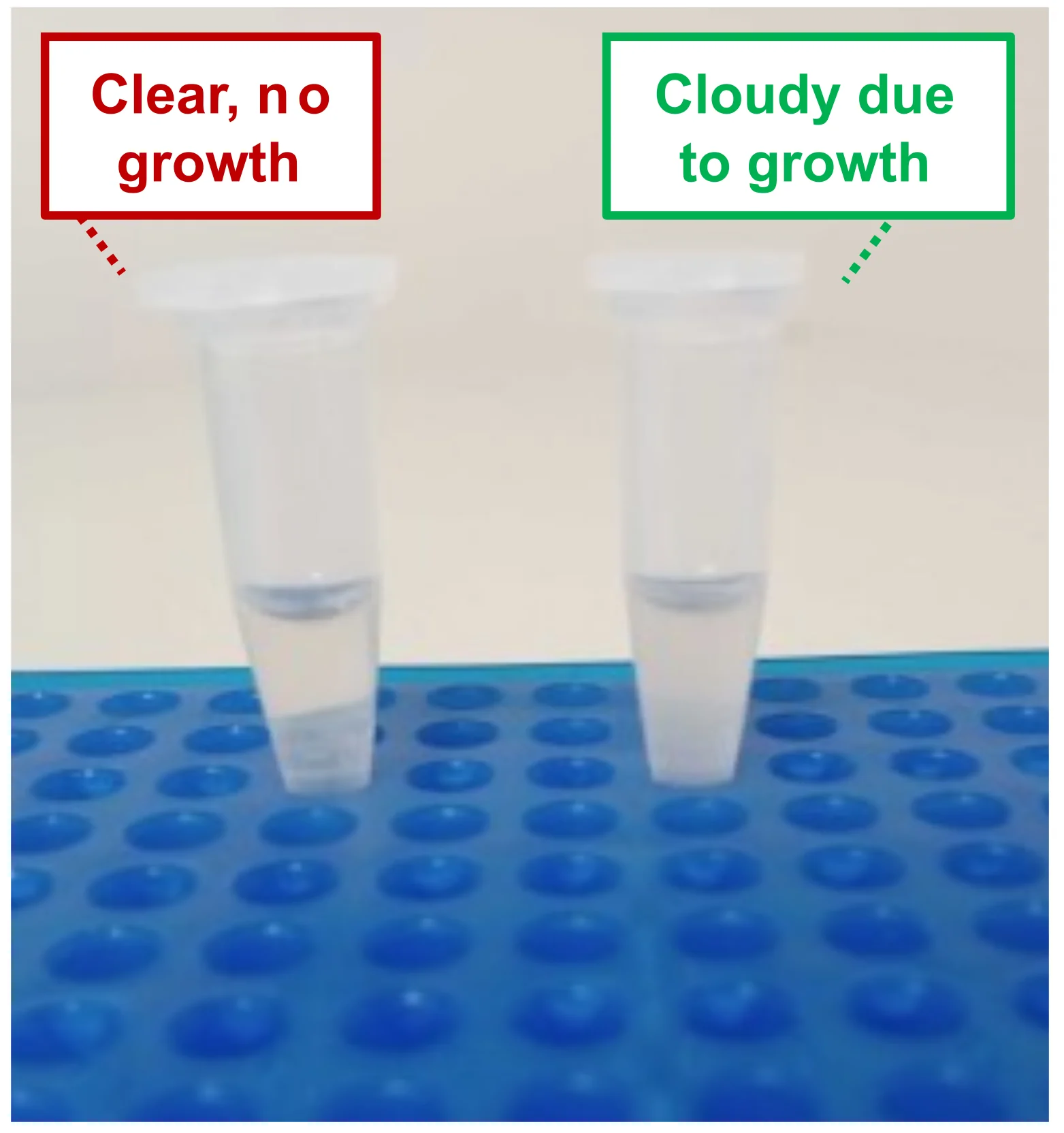
An example photo showing successful bacterial culture growth, compared to no growth. The image shows two 1.5 mL tubes with (left) 500 μL of standard LB (i.e., no bacteria) and (right) 500 μL of *E. coli* MG1655 *bioB::kan* grown in standard LB using the methods in this section. Successful bacterial growth is indicated by the increased turbidity of the culture relative to the LB-only control, which remains transparent and allows clear visualization of the blue tube rack positioned behind the tubes. The bacterial culture was grown by shaking LB inoculated with MG1655 at 37 °C for 16 h.

1. Initially, in sterile conditions (e.g., in an operating biosafety cabinet), transfer a colony from the LB agar plate to a 50 mL centrifuge tube containing 25 mL of LB using a manual pipette and a 200 μL unfiltered pipette tip.

2. For *E. coli* MG1655 *bioB::kan*, supplement each 25 mL aliquot of LB with 25 μL of 50 mg/mL of kanamycin (i.e., a 1:1,000 dilution) to grow the Kan-resistant bacteria.

3. Repeat **steps A1–2** for each bacterial strain until at least three colonies are prepared in their own 50 mL centrifuge tubes. This should be done in case the colonies from **steps A1–2** do not grow during the overnight incubation in the next step—you need only one “good” tube (see next step for explanation).

4. Incubate 50 mL tubes containing bacterial colonies in LB in an orbital shaker set to 37 °C and ~300 rpm shaking overnight (16–18 h). The resulting bacterial cultures should appear cloudy, indicating successful bacterial growth (**Figure 1**). Proceed only with successful cultures.

**(Day 1)**

5. Subsequent bacterial cultures can be made by diluting 5 μL of liquid culture into 25 mL of fresh LB (selective or non-selective) instead of a colony from an LB agar plate and then repeating **step A4**. Culture 150 mL of *E. coli* MG1655 *bioB::kan* for one biological replicate of this experiment.

**Critical:** Ensure that sufficient bacterial cultures have been generated at least one day before NGM plates for nematode culturing need to be seeded (*seeding* is defined below).

*Notes:*

*1. Store all LB agar plates and liquid bacteria in a refrigerator set to 4 °C. LB agar plates can be used within three months. Use liquid bacteria within three weeks.*

*2. Glycerol stocks of bacteria should be generated for long-term (years) storage. To do this, add 500 μL of liquid bacteria to 500 μL of 50% (v/v) glycerol in nuclease-free water in sterile conditions. Keep the diluted bacterial solution in an ultracold (e.g., -80 °C) freezer. This glycerol stock can be plated onto fresh LB agar plates to regrow bacterial colonies.*

**B. Making agar plates for *C. elegans* culturing, biotin treatment, and chemotaxis assays**

**Figure 2.**
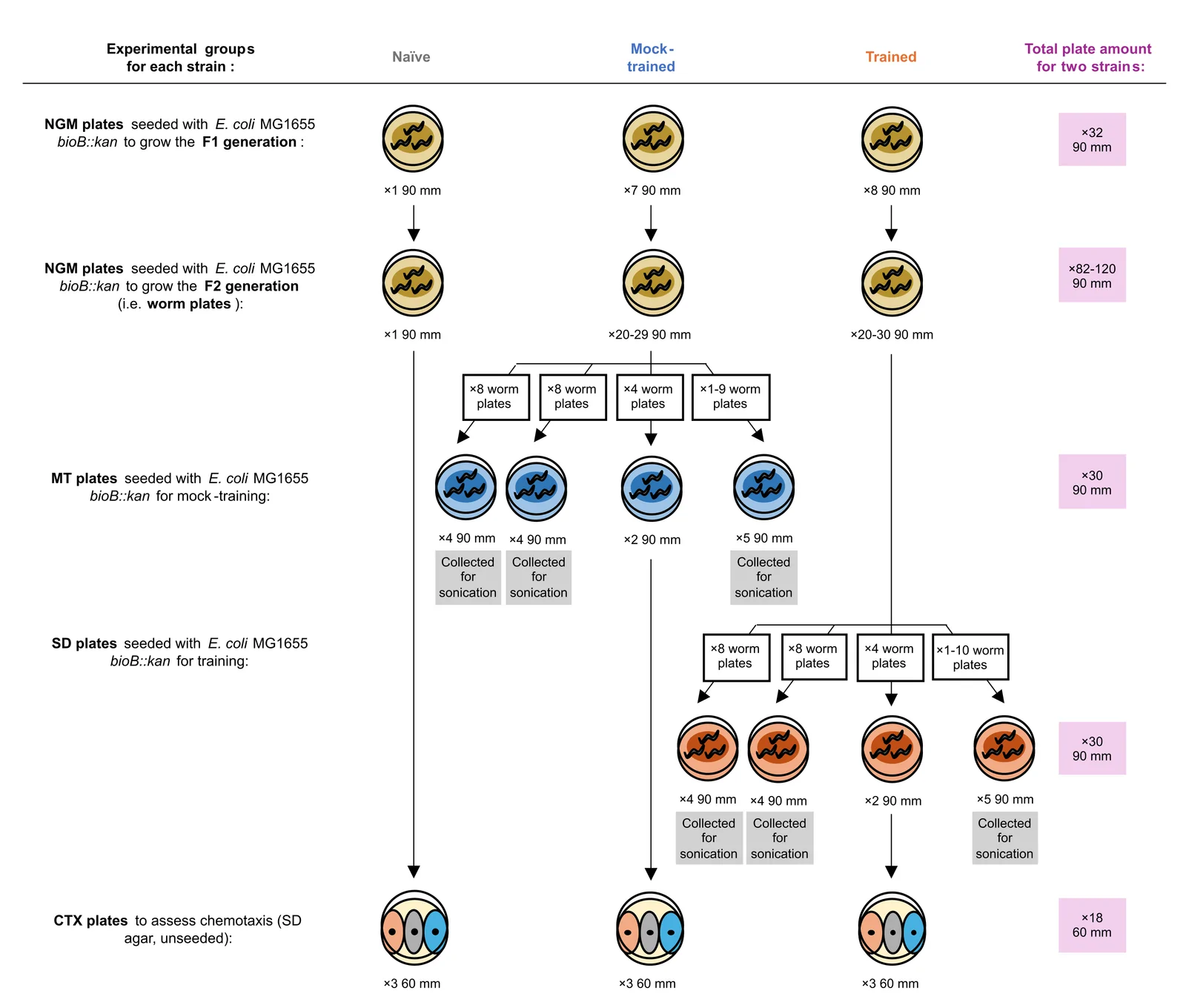
Agar plates required for each experimental group. This schematic illustrates the workflow and distribution of animals across experimental groups for a single strain and a single biological replicate. Plate numbers are shown in the format "number of plates × plate diameter." Petri dishes are prepared with 10 (60 mm) or 20 mL (90 mm) of agar per plate. Animals are depleted of biotin for two generations prior to experimentation, and naïve groups consist of animals that do not undergo mock-training or training. The schematic is intended for illustrative purposes. Plate numbers shown within the workflow correspond to a representative experiment for a single strain. Those on the right (in pink) represent plate numbers required for the standard experimental design described in this protocol, which includes both the non-transgenic control strain (N2) and the TurboID-expressing strain (YLC207). Additional worm plates are typically prepared to allow exclusion of contaminated or otherwise unsuitable plates (see General note 1) while ensuring sufficient material for downstream proteomic analyses. In our hands, 20–30 × 90 mm worm plates per experimental group are generally sufficient. Animals are typically processed in batches of up to 8 × 90 mm worm plates at a time for the technical reasons described in the main text.

Multiple types of agar plates are necessary for this protocol, which differ based on agar composition and bacteria used during seeding (defined below). This protocol is separated by days since different agar plate types have differing expiry periods. NGM agar is prepared on **day 1** and seeded on **days 2 and 5**. Other agar types are dispensed into Petri dishes on **day 6**, with agar plates used to mock-train or train animals seeded on **day 8. Figure 2** provides an overview of the required volumes of agar to prepare for each strain per biological replicate, as well as the total number of plates required to generate each experimental group for downstream proteomic experiments. Storage conditions for each agar plate type are provided in the steps below.

**(Day 1)**

1. Prepare 3,500 mL of NGM (see Recipes). To do this, autoclave the powders in ultrapure water in borosilicate bottles, cool agar in a water bath to 55 °C for 15 min, and then add solutions in sterile conditions.

2. Use a peristaltic pump (set to 20 mL) to prepare 136 plates (90 mm diameter) with NGM agar.

*Notes:*

*1. We recommend using a peristaltic pump to ensure that the volume of agar is fixed between biological replicates for NGM plates used to culture* C. elegans.

*2. NGM plates of 136 × 90 mm diameter are sufficient to culture animals for one biological replicate of this experiment.*

*3. Excess agar can be dispensed into 60 mm Petri dishes (10 mL agar) for* ***section C***.

3. Incubate NGM agar plates from **step B2** overnight (~16 h) at room temperature, to allow agar to set (see General note 2).

*Note: NGM agar plates can be prepared in advance before* ***day 1***, *so long as they are used for this protocol to culture* C. elegans *within one month. Store unseeded NGM agar plates in airtight boxes in a 4 °C refrigerator (570 L) or cold room before use (see* ***step B4*** *for the definition of seeding).*

**(Day 2)**

4. Add 250 μL of *E. coli* MG1655 *bioB::kan* to each Petri dish (90 mm diameter) containing NGM agar, for 16 NGM agar plates. Afterward, swirl the plate by gently moving it in a circular motion to spread out the liquid bacteria into a thinner layer. This must be done in an operational biosafety cabinet to minimize contamination.

*Notes:*

*1. We refer to the process of pipetting bacteria, the food source for* C. elegans, *onto agar plates as “seeding” plates.*

*2. Bacteria should be dispensed onto the center of an agar plate. Ideally, the bacteria should not touch the edge of the plate, as this often leads to worms crawling off the agar, reducing the number available for downstream experiments.*

5. Allow bacteria to dry by incubating the 16 seeded plates overnight (~24 h), agar side down, at room temperature in a biosafety cabinet (see General note 2).

*Note: These plates will be used to decrease biotin levels in progeny* C. elegans, *from parent animals that have never experienced biotin depletion previously (i.e., referred to as the F1 generation in* ***Figure 2*** *). Parent animals will be transferred onto these plates on* ***day 4***. *These plates must be stored at room temperature and used within 48 h.*

6. Seed excess 60 mm diameter plates (from **step B2, note 3**) with 100 μL of *E. coli* OP50 per plate, by repeating **steps B4–5**.

*Note: These plates serve to maintain a stock of each* C. elegans *line containing animals that have not undergone biotin depletion. They can be stored in airtight boxes in a 4 °C refrigerator (570 L) or cold room and used within two months. Smaller diameter plates can be used since two worms at a minimum are required to maintain each line.*

**(Day 5)**

7. Repeat **steps B4–5** to seed 120 × 90 mm diameter NGM agar plates with *E. coli* MG1655 *bioB::kan*.

*Note:* C. elegans *that undergo behavioral experiments in* ***sections D and E*** *will be cultured on these plates (see F2 generation in* ***Figure 2***).

**(Day 6)**

8. Prepare 30 × 90 mm diameter plates with salt-deficient SD agar (for training), 20 × 60 mm diameter plates with SD agar (for chemotaxis assays, called CTX plates), 30 × 90 mm diameter plates with mock-training MT agar (for mock training), and 5 × 60 mm diameter plates with salt gradient SG agar (see Recipes), based on **steps B1–3**.

*Notes:*

*1. Ensure that you label Petri dishes before adding agar, so you can visibly differentiate between agar plate types.*

*2. SD, MT, and SG agar should be dispensed into Petri dishes immediately (i.e., no cooling is required after autoclaving).*

*3. SD and MT agar plates for training/mock training must be stored with agar side down in a biosafety cabinet at room temperature and used within five days (see General note 2).*

*4. CTX and SG plates can be stored in airtight boxes in a 4 °C refrigerator (570 L) or cold room and must be used within two months. SG plates are needed to form salt concentration gradients on CTX plates (see* ***day 11*** *in this section). These plates will be used to quantify salt chemotaxis behavior on* ***day 12*** *(see* ***section E*** *); it is therefore crucial that the variables used to construct the environment that aims to test memory retrieval are consistent. The lower temperature will reduce the rate of agar desiccation [35].*

9. Prepare WWB, NSB, and modified LB solutions (see Recipes).

*Note: WWB and NSB can be stored at room temperature, whereas modified LB should be kept in a refrigerator (570 L) set to 4 °C as a short-term storage location. These solutions should be used within three weeks.*

**(Day 8)**

**Steps B10–14** outline steps to wash bacteria before seeding SD and MT plates for training/mock-training. This washing replaces the standard LB media used to grow bacteria with modified LB to (i) minimize the sodium chloride content that animals are exposed to during training, as sodium chloride from standard LB can hinder salt associative learning, and (ii) provide enough starting bacteria and nutrients for sufficient lawn growth.

10. Add 1.5 mL of *E. coli* MG1655 *bioB::kan* per tube in 20 autoclaved 2.0 mL centrifuge tubes. Use the bacteria cultured during **step A5**.

11. Centrifuge *E. coli* MG1655 *bioB::kan* aliquots (11,000 rcf, 30 s, 22 °C).

*Note: A visible pellet should be at the bottom of each centrifuged tube.*

12. Remove supernatant from centrifuged *E. coli* MG1655 *bioB::kan* aliquots. Add 750 μL of modified LB to the pellet.

13. Mix bacteria with modified LB by gentle pipetting before transferring to a new 50 mL centrifuge tube.

14. Repeat **steps B10–13** twice using the same 2 mL centrifuge tubes. Resuspend the concentrated *E. coli* MG1655 *bioB::kan* in 46.5 mL of modified LB for seeding. Add 465 μL of freshly made 0.1 M biotin to the concentrated liquid bacteria (1 mM final concentration) [25].

*Note: This final concentration was chosen for biotin because it has been used previously to perform proximity labeling in* C. elegans *neurons with the TurboID enzyme [25]. Moreover, it was compatible with the duration we required for proximity labeling. To explain, it needed to be the entire time window that animals were trained to learn (i.e., on solid media for 6 h), as our aim was to label as many proteins present in their neurons during this learning period. This compatibility was validated by visualizing biotinylated proteins on a western blot, from animals exposed to 1 mM biotin on solid media for 6 h (see* ***Validation of protocol*** *for details) [14]. We suggest users initially perform western blots in pilot experiments to confirm that the enzyme can sufficiently label proteins during the biological process of interest. Any issues arising from this are likely solvable by adjusting the final biotin concentration. More troubleshooting information can be found in the comprehensive protocol [26].*

15. In a biosafety cabinet, seed 30 MT agar plates (90 mm diameter) and 30 SD agar plates (90 mm diameter) with 750 μL of *E. coli* MG1655 *bioB::kan* in modified LB supplemented with biotin per plate (see General note 2 for storage instructions until **day 12**).

**(Day 11)**

16. Discard CTX and SG plates with unexpected fungal and/or bacterial contamination (see General note 1).

*Note: One SG agar plate and nineteen CTX plates are the minimum amount required for this protocol (see* ***Figure 2*** *for details).*

17. Bring at least 19 CTX plates and 1 SG agar plate to room temperature by incubating on the bench for 3 h in an airtight box.

*Note: You can prepare one additional CTX and SG plate for this step, as a backup. This step should be done 18 h before you begin* ***section D***.

**Figure 3.**
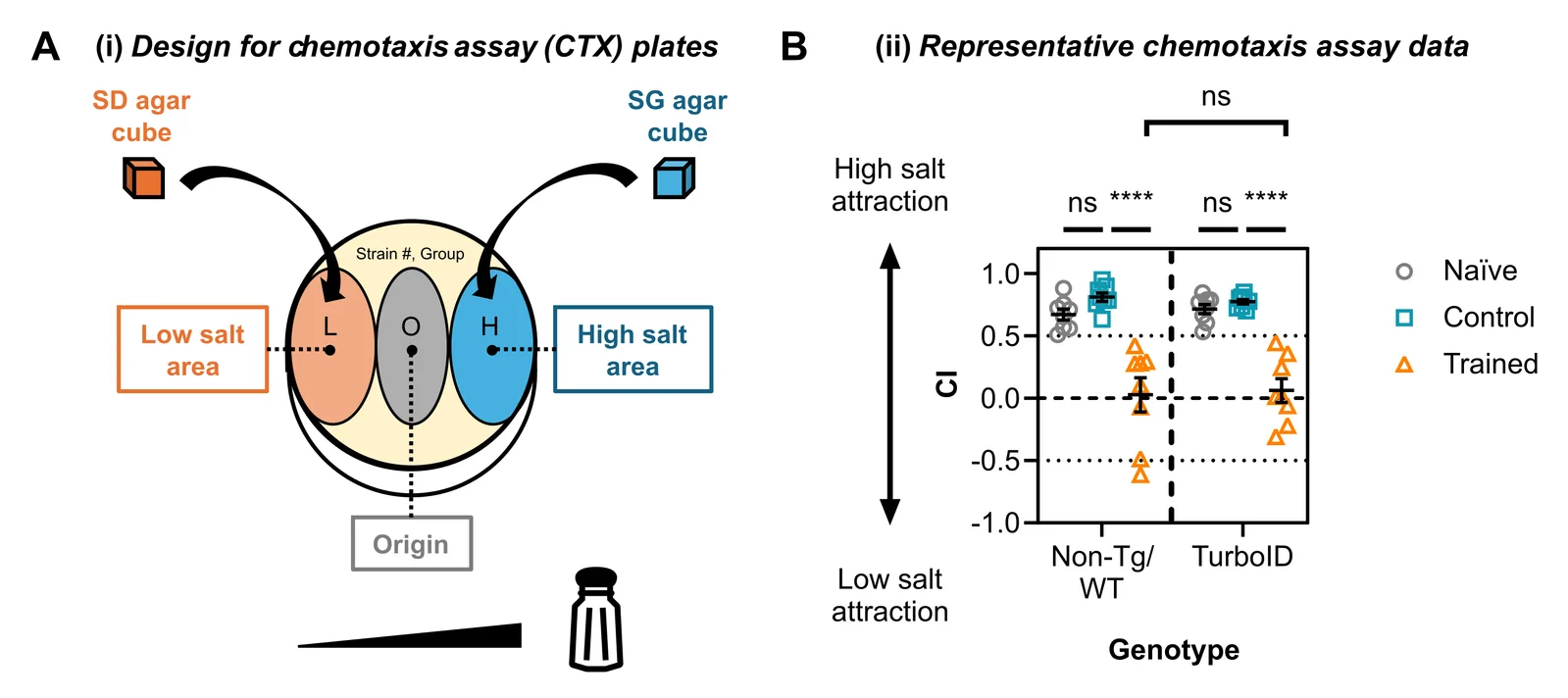
Representative chemotaxis assay (CTX) plate design and corresponding behavioral data. (A) Design for CTX plates. This image represents labels drawn onto the bottom of a CTX plate (60 mm diameter). Each CTX plate is defined by three regions shown above, from left to right: (i) low salt (diameter = 24 mm; in orange), the origin (to transfer worms onto for assays; 20 mm short axis and 24 mm long axis; in grey), and (iii) high salt (diameter = 24 mm; in blue). The center of each region is marked with an appropriate indicator (i.e., L for low salt, O for origin, and H for high salt). CTX plates are also labeled with the strain number and experimental group that will be tested on the plate, before animals are transferred onto them for assays. Salt gradients are generated by overnight incubation with one cube of SD agar (with no salt) and one cube of SG agar (containing high salt) per CTX plate. A photo showing a labeled CTX plate with SD and SG agar cubes is available in our eLife paper (see **Figure 1, figure supplement 2** in [14]). **(B)** Representative CTX data. Non-transgenic/non-Tg animals from the N2 wild-type/WT line were compared to TurboID-expressing *C. elegans* from strain YLC207. Each data point represents a chemotaxis index (CI) value from one biological replicate (n = 8), averaged from three technical replicates (26–260 worms/technical replicate). Statistical analysis: Two-way ANOVA and Tukey’s multiple comparisons test (****p ≤ 0.0001; ns = non-significant). Data represented by mean ± SEM. These figures were adapted from **Figure 1B** and **Figure 1, figure supplement 2** from [14].

18. We recommend creating a reusable template to consistently label the underside of CTX plates for chemotaxis assay experiments using a transparent plastic sheet. The template should have one spot at the center (as the “origin,” labeled with the letter O), and the remaining spots should be equidistant from each other and 5 mm from the plate edge. One spot near the plate edge should be labeled “L” (as “low salt”) and the other with “H” (as “high salt”) (**Figure 3A**). Labels can be made with a permanent marker pen.

*Note: Three circles should be drawn with the spots mentioned above as their centers. Circles around L and H spots should have a 2.4 cm diameter, and the O circle should have a 2.0 cm short axis and 2.4 cm long axis (* ***Figure 3A***).

19. Use a Kimwipe to remove excess condensation from the outside of each CTX plate.

20. Use a marker (1.0 mm) to label the underside of each CTX with three spots and circles using the CTX plate labeling template. This can be done by laying a CTX plate onto the top of the template while it is agar side up.

21. Each experimental group assayed for salt chemotaxis in this section will require three CTX plates. Label six plates each as naïve, mock-trained, and trained. Within each condition, label with the strain identifier, e.g., three plates as N2 and three as YLC207. There will be 18 labeled CTX plates in total; leave one as a spare (see **step B24** onward).

*Note: One CTX plate represents one technical replicate of chemotaxis assay data for a specific group.*

22. Prepare labeled CTX plates by (i) laying them agar side down with lids next to each dish and (ii) removing excess condensation from the inside edge of each lid using Kimwipes. This is important as the next steps need to be done quickly.

23. Clean tweezers with 80% (v/v) ethanol and one Kimwipe before use in subsequent steps, to minimize contamination.

24. Use a new razor blade to cut out 5 × 5 mm cubes of SD agar from the spare unlabeled CTX plate, and then carefully transfer one SD agar cube onto each CTX plate, centered on top of the spot labeled “L,” using clean tweezers.

25. Gently press the top side of each SD agar cube into the CTX plate with tweezers to maximize contact between the SD agar cube and the surface of the SD agar in each CTX plate.

26. Repeat **steps B24–25** with a room-temperature SG agar plate (from **step B17**), centering SG agar cubes onto the spots labeled “H.”

27. Replace lids onto each CTX plate and then store CTX plates in an airtight box on the top shelf of an incubator (300 L) set to 22 °C for use in **section E** during **day 12**.

*Notes:*

*1. This will allow salt concentration gradients to form on the surface of the CTX plates.*

*2. Plates should be fully prepared and ready for storage 15 h before beginning biotin treatment (* ***section D***).

**C. Depleting biotin levels in *C. elegans* as preparation for protein proximity labeling**

*C. elegans* undergo biotin depletion for two generations for this protocol. Parent animals (fed *E. coli* OP50 during development) are used on **day 4** to grow progeny with *E. coli* MG1655 *bioB::kan* as their food source. This biotin-auxotrophic strain is also fed to the second (F2) generation, with cultures prepared on **day 7**. F2 animals will constitute experimental groups for behavioral experiments in **sections D and E**.

**(Day 4)**

1. Transfer egg-laying (day 1) adult hermaphrodites from maintenance plates (NGM agar with *E. coli* OP50 in standard LB) onto 8 × 90 mm NGM agar plates with *E. coli* MG1655 *bioB::kan* in standard LB (see General note 3). For N2, we transfer 6 worms per plate; for the YLC207 line, we transfer 4 worms per plate (see General note 4).

*Notes:*

*1. Animals from line YLC207 express TurboID in all neurons; they will be used to label proteins present during mock-training or training. As YLC207 contains the TurboID transgene as an extrachromosomal array, transgenic animals should be selected using a fluorescence microscope.*

*2. N2 functions as a TurboID-negative control.*

2. Incubate transferred worms for three days at 22 °C (or equivalent) to allow for the production and growth of progeny to the L4 developmental stage on NGM agar plates seeded with *E. coli* MG1655 *bioB::kan.*

*Note: Animals cultured during this step serve as the F1 generation.* E. coli *MG1655 bioB::kan is a biotin auxotrophic bacterial strain, so F1 animals will feed on the bacteria and subsequently lower their biotin levels (relative to their parent generation).*

**(Day 7)**

3. Deplete biotin levels for the F2 generation of nematodes by repeating **steps C1–2** with modifications. Use **F1 L4 hermaphrodites** (i.e., progeny obtained from **step C2**) to generate the F2 cohort. Prepare a total of 120 × 90 mm diameter NGM agar plates as follows:

a. N2 strain: 360 animals distributed as 6 worms per plate across 60 plates.

b. YLC207 strain: 240 animals allocated as 4 worms per plate across 60 plates.

*Note: To ensure that both strains reach the correct developmental stage at the time of biotin treatment (* ***day 12*** *), set up these cultures in a staggered manner. Ideally, initiate one strain in the morning and the other in the afternoon. This will allow one population to be ready for biotin treatment in the morning (called “Strain 1” below), while the second population reaches the appropriate developmental stage in the afternoon of the same day (henceforth called “Strain 2”).*

4. Store the 120 agar plates for five days at 22 °C to grow animals that are completely biotin-depleted, for **sections D and E**.

*Note: NGM plates containing* E. coli *MG1655 bioB::kan and F2 animals will henceforth be referred to as “worm plates.”*

**D. Biotin treatment during the training phase of salt associative learning**

In this section, two strains are transferred from worm plates onto MT and SD plates seeded with MG1655 for biotin treatment. One strain is wild-type and does not express TurboID (from the N2 line), and the other line is the TurboID-expressing transgenic strain YLC207. From this, four experimental groups are prepared: (i) mock-trained from Strain 1 (in **steps D3–16**), (ii) trained for Strain 1 (**step D17**), (iii) mock-trained from Strain 2 (**step D18**), and (iv) trained for Strain 2 (**step D18**). Animals must be treated quickly and consistently for groups to be comparable, but there is a limit to how many worm plates can be handled simultaneously, so each group must be prepared one at a time. This protocol provides steps to prepare each group in the order provided in the list above, but note that this does not need to be fixed between biological replicates.

Our protocol involves handling four groups (outlined above) and then collecting enough biotin-treated animals from each group in one biological replicate for downstream proteomics. To summarize, for each group, 8–13 × 100 μL *C. elegans* pellets are collected from 20–30 worm plates for distribution on 10–15 × 90 mm MT or 10–15 × 90 mm SD plates. Animals are then collected into 2–3 × 1.5 mL tubes containing ~100 μL of *C. elegans* per tube (annotated as grey text boxes in **Figure 2**). Typically, 150–300 μL of *C. elegans* is sufficient to extract the required protein from one group and biological replicate in this workflow (i.e., ~1 mg; **section F** details protein extraction protocols).

An important note is that this section takes ~13 h. This accounts for users transferring animals within one group, while other groups undergo incubations to save time. An alternative strategy could be to pool *C. elegans* from multiple biological replicates. **Sections A–E** could be repeated several times, with a smaller number of worm plates per biological replicate to constitute each biotin-treated group. However, this may not be suitable in the instance that limiting potential variability within biological replicates is essential (e.g., due to high background). We recommend that users consider these concerns when planning to perform this experiment.

**(Day 12)**

1. Assess each worm plate for the following variables: (1) the state of the food lawn, (2) the presence of any fungal or bacterial contamination, (3) the developmental stage of worms on each Petri dish, and (4) the presence of male animals. Discard plates with compromised food lawns, contamination, and/or males (see General note 1).

*Notes:*

*1. Animals must be at the correct developmental stage. For example, young adult hermaphrodites were used in [14]. We only used worm plates that contained ~99% of animals at the adult stage. This can be adapted in the instance that you aim to analyze proteomes from differently aged animals.*

*2. Males can occur spontaneously (0.1%) from hermaphrodite parents that undergo self-fertilization to generate progeny [36].* C. elegans *have been reported to display different gene expression patterns based on their sex [37], so it is essential that males are not included in the animal population that undergoes biotin treatment for downstream proteomic experiments.*

*3. Ideally, you would use 29–30 worm plates for each mock-trained or trained experimental group, but this number will likely decrease after discarding Petri dishes that do not satisfy the criteria outlined above. Therefore, note that the experiment can proceed with a minimum of 20 worm plates. In the instance that you have fewer than 20 worm plates for an experimental group for biotin treatment, you will not have enough protein to proceed with the subsequent steps.*

2. Discard any MT and SD plates seeded with MG1655 that display fungal or bacterial contamination (see General note 1).

*Notes:*

*1. MT plates will function to mock-train animals by exposing them to salt and food, which does not change their untrained behavior toward salt (versus untrained/naïve animals, see* ***Figure 3B*** *). Biotin is added to these plates to identify the mock-trained proteome in neurons, as a learning-negative control.*

*2. Animals will be trained to associate no salt with food on SD plates. This learned association is represented by a decrease in attraction to salt concentrations experienced during cultivation (vs. naïve animals, see* ***Figure 3B*** *). Training is done in the presence of biotin to label proteins in* C. elegans *neurons during learning.*

3. Preparation of mock-trained cohort for Strain 1 (e.g., wild-type no-TurboID strain): Select 8 worm plates for strain 1 as one batch that will be prepared for biotin treatment.

*Note:* ***Steps D5–10*** *involve washing animals three times within 8 min per batch, before adding them to MT plates for mock-training and biotin treatment. It is recommended to handle animals from a maximum of 8 worm plates simultaneously as a single batch for an experimental group, due to the need to minimize the time that animals are in solution (see General note 5). This serves to remove MG1655 bacteria from worm plates with no biotin before incubation on MT plates, so that worms only consume food containing biotin on MT plates during mock-training.*

4. Label MT plates with the strain number (e.g., N2) and experimental group type (i.e., mock-trained). In addition, add a label that can be used to identify the batch for which the animals on the plate belong. For example, label MT plates with “#1” so you can identify the first batch of MT plates prepared for the biotin treatment experiment. This is important so that you can keep track of how long animals in each batch have been exposed to biotin, which needs to be consistent to minimize variation within worm populations that constitute an experimental group.

5. **Wash #1**: Sequentially wash 2 worm plates once, until animals from 8 plates are in four 15 mL centrifuge tubes. For 2 worm plates, (i) add 4 mL of WWB to the center of one plate using a serological pipette controller and glass serological pipette tip (as a “clean” tip), (ii) slightly tilt the worm plate with WWB toward you (~45° angle) pipetting up the WWB containing worms (with a new tip, as the “dirty tip”; see rationale in **step D16, note 1**), and then dispensing the solution above the food lawn to gather as many worms as possible in solution, (iii) pipette up *C. elegans* in WWB from the first plate, dispense onto the center of the second NGM plate, and collect these worms in the same way, and then (iv) transfer animals in WWB to one 15 mL tube.

6. Set a timer for 2 min immediately after collecting animals from the first two worm plates.

*Notes:*

*1. For* ***sections D and E***, *keep all four 15 mL tubes containing worms in solution upright by using a centrifuge tube rack, so that animals can settle into a pellet by gravity.*

*2. Wash #1 should take approximately 45 s maximum for two worm plates (see General note 5).*

*3. It is expected to have 100–200 μL of adult hermaphrodite* C. elegans *per 15 mL tube.*

7. **Wash #2**: Sequentially replace supernatant in each 15 mL tube from **step D5** with 1 mL of clean WWB.

*Notes:*

*1. Ensure that you pipette up carefully so that you do not disturb worm pellets when removing supernatant containing bacteria, to maximize sample size for the biotin treatment experiment.*

*2. When you dispense WWB onto each worm pellet, this should be done gently for three seconds.*

*3. Perform this step in the same order that you prepared worm pellets for each 15 mL tube in* ***step D5***.

8. Use a timer set to 1 min as soon as you replace the supernatant for the first 15 mL tube in **step D7**, to keep track of time. Wash #2 should be done for each worm pellet within 30 s.

9. **Wash #3**: Repeat **steps D7–8** from this section with 1 mL of WWB per 15 mL tube.

10. Remove the clear supernatant from each 15 mL tube containing worms in the same order as animals were collected in **step D5**, leaving approximately a 1:1 volume ratio of *C. elegans* and WWB in each tube.

11. Gently mix animals in each 15 mL tube by pipetting up-and-down once, and then immediately but carefully distribute animals across four aliquots (≤1 cm in diameter) dispensed next to the food lawn on an MT plate.

12. Repeat **step D11** until all worm pellets are transferred onto individual MT plates.

13. Immediately wick excess WWB from each droplet on MT plates (see General note 6).

*Note: The entire process outlined in* ***steps D10–13*** *in this section should take ≤2 min.*

14. Transfer one batch of MT plates containing wicked animals onto the top shelf of an incubator (300 L) set to 22 °C. Ensure the plates are not disturbed for the duration of the mock-training (6 h).

15. Set a timer for 6 h to track the progress of mock-training for animals on MT plates (from **step D14**).

16. Repeat **steps D3–15** for the remaining 21 NGM plates containing worms until all 15 MT plates are incubating at 22 °C.

*Notes:*

*1. You can use the same glass serological pipette tips for wash steps for all 29 NGM plates containing the same* C. elegans *line for the same experimental group, so long as you ensure the clean tip is not used to transfer animals.*

*2. The same 15 mL tubes can be utilized to wash animals with the same strain and experimental group.*

17. Preparation of trained cohort for Strain 1 (e.g., wild-type no-TurboID strain): Repeat **steps D3–16** to train animals from 30 worm plates sharing the same *C. elegans* line, with modifications. Use NSB to wash animals for **step D9** (not WWB) and salt-deficient SD plates for biotin treatment (instead of MT plates).

*Note: Using NSB depletes salt levels from the solution containing worms to be transferred onto SD plates.*

18. Preparation of mock-trained and trained cohorts for Strain 2 (e.g., TurboID strain): Perform **steps D3–17** for the second *C. elegans* line in the afternoon, so that animals can undergo mock-training and training.

*Note: For each line, animals from 2 MT and 2 SD plates will be used to assess salt chemotaxis in* ***section E*** *on* ***day 12***. *The remaining MT and SD plates will be used in downstream proteomic experiments (* ***section F***).

**E. Behavioral assessment of learning by salt chemotaxis assay**

These steps aim to assess how animals from two strains respond to salt when naïve/untrained, mock-trained with salt and food, or trained with no salt and food. This protocol outlines the preparation for each experimental group in the following order: (i) naïve from Strain 1 (from 1 worm plate, in **steps E2–11**), (ii) naïve from Strain 2 (**step E12**), (iii) mock-trained from Strain 1 (2 MT plates with food and worms, **steps E13–17**), (iv) trained for Strain 1 (2 SD plates with food and worms, **step E18**), (v) mock-trained from Strain 2 (**step E19**), and (vi) trained for Strain 2 (**step E19**). See **Figure 3B** for chemotaxis assay data.

Chemotaxis assays are prepared for naïve animals during 6-h incubations for mock-training and training. The order in which naïve animals are transferred onto CTX plates on **day 12** is not fixed.

The remaining groups must be prepared in the same order as in **section D**. One batch from each mock-trained and trained group is required to assay chemotaxis behavior. This means that (i) animals are collected for chemotaxis assays after the 6-h biotin treatment period is complete for that batch, and (ii) these steps are done during 6-h incubations for other batches.

**(Day 12)**

1. Prepare 100 μL of 0.1 M NaN_3_ solution fresh on **day 12** to use on the same day. Store this solution on wet ice, on the lab bench where you will transfer worms onto CTX plates.

2. Chemotaxis assay of naïve worms from Strain 1: Subsequent steps involve CTX plates prepared with salt gradients on **day 11** (see **section B** for details). Put prepared CTX plates labeled with the same strain number and “Naïve” onto the lab bench, by placing them agar side down with lids next to their dishes. Remove any condensation on plate lids using Kimwipes.

3. **Wash #1**:

a. Add 3 mL of WWB to the center of 1 × worm plate using a serological pipette and glass tip.

b. Slightly tilt the plate with WWB toward you (~45° angle), pipetting up the WWB containing worms, and then dispensing the solution above the food lawn to gather as many worms as possible in the solution.

c. Transfer animals in WWB to one clean 15 mL centrifuge tube positioned upright in a centrifuge tube rack.

d. Set a timer for 80 s to allow animals to pellet by gravity; 50–100 μL of adult hermaphrodite *C. elegans* are expected to be present in each 15 mL tube (see General note 5).

4. During the 80-s incubation period in **step E3**, use tweezers to dispose of SD and SG cubes from naïve CTX plates prepared during **step E2**.

5. **Wash #2**: Replace supernatant in each 15 mL tube with 1 mL of clean WWB. Slowly dispense WWB onto each worm pellet for 3 s, and then set a timer for 30 s. Pipette carefully so that you do not disturb the worm pellets when removing supernatant.

6. During the 30-s incubation time in **step E5**, add 1 μL of 0.1 M NaN_3_ to L and H spots on each naïve CTX plate prepared during **step E2**.

7. Remove the clear supernatant from each 15 mL tube containing worm pellets until there is an approximately 1:1 volume ratio of *C. elegans* and WWB.

8. Gently mix animals in each tube by pipetting up and down once using a filtered low-retention 200 μL pipette tip. Then, immediately but carefully distribute animals across three aliquots ≤1 cm in diameter, dispensed on the O spot on each CTX plate. Immediately wick excess WWB from each droplet using a Kimwipe (see General note 6); this process should take ≤2 min.

9. Incubate CTX plates containing wicked animals on the top shelf of an incubator (300 L) set to 22 °C for 45 min. Position CTX plates agar side down in plastic bunding (e.g., an airtight storage box without the lid). This 45-min period will allow animals sufficient time to display their behavioral response toward the salt concentration gradient. Ensure the plates are not disturbed during chemotaxis.

10. Set a timer for 45 min.

11. After the 45-min incubation is over, CTX plates should then be stored agar side up in a refrigerator (570 L) set to 4 °C, to be addressed on **day 13**.

12. Chemotaxis assay of naïve worms from Strain 2: Repeat **steps E2–11** to assay naïve salt chemotaxis of the second *C. elegans* line used in this experiment.

13. Chemotaxis assay of mock-trained worms from Strain 1: ≤15 min before a 6-h incubation period finishes for one batch of mock-trained animals, prepare CTX plates as in **step E2**.

14. **Wash #1**: When the 6-h incubation period is completed for one batch of mock-trained animals, repeat **step D5** to transfer mock-trained animals with the same worm line and batch from 2 MT plates into one clean 15 mL tube. Set a timer for 80 s to allow animals to pellet by gravity.

*Note: 100–200 μL of adult hermaphrodite* C. elegans *are expected to be present in each 15 mL tube.*

15. **Wash #2**: Repeat **steps E5–7** (see General note 5). Proceed with **steps E8–11** for one worm pellet from a single batch, to assess chemotaxis for mock-trained animals from Strain 1.

*Note: One worm pellet is sufficient to assess salt chemotaxis for mock-trained animals for Strain 1 per biological replicate. The remaining pellets are used to prepare proteomics samples for mass spectrometry.*

16. Perform **steps E14 and E15** for the remaining batches of the same experimental group. However, for **step E15**, combine pellets of washed mock-trained *C. elegans* from Strain 1 in a new 1.5 mL centrifuge tube on ice (i.e., instead of transferring animals onto CTX plates). This means that worm populations from each batch are in individual 1.5 mL tubes. This can be done with a manual pipette and a filtered low-retention 200 μL pipette tip. Incubate these tubes on ice for ≥6 min to allow animals to sediment into tightly formed pellets.

17. Remove the supernatant from 1.5 mL tubes containing *C. elegans* with a manual pipette and then store tubes in an ultracold -80 °C freezer.

*Note: Animals can be stored in the freezer for up to 6 months, for use in* ***section F***.

18. Chemotaxis assay of trained worms from Strain 1: Perform **steps E13–17** for trained animals on SD plates.

19. Chemotaxis assay of mock-trained and trained worms from Strain 2: Repeat **steps E13–18** from the second *C. elegans* strain for both mock-trained and trained cohorts. At the end, you have four groups (mock-trained + no TurboID, trained + no TurboID, mock-trained + TurboID, and trained + TurboID), each having undergone two washes, with some worms used either for a chemotaxis assay to assess their learned behavior or frozen at -80 °C for later proteomics sample preparation.

**(Day 13)**

20. Assessing chemotaxis behavior: Set up a stereomicroscope. We recommend counting worms on CTX plates with the agar side up for ease of marking counted worms.

21. Remove excess condensation with a Kimwipe from the labeled side of the CTX plate containing worms.

22. Use a clicker counter to manually count the number of worms in each region of the CTX plate. These regions are marked as circles and “L” (low salt region), “O” (origin), and “H” (high salt region). Use a fine-tip marker to mark which worms have been counted already on the bottom side of the plate.

*Note: Exclude CTX plates containing fewer than 20 animals, as this is an insufficient number of animals to assess salt chemotaxis from a worm population.*

23. Repeat **step E22** for worms outside of the low salt, origin, and high salt regions, so that you can calculate the chemotaxis index (CI) for that CTX plate, as follows:

$$CI=\frac{\left(\#\;Worms\;in\;the\;high\;salt\;region\;-\#\;Worms\;in\;the\;low\;salt\;region\right)}{\left(\#\;Total\;worms\;on\;the\;CTX\;plate\;-\#\;Worms\;in\;the\;origin\;region\right)}$$

*Note: CIs serve to quantify chemotaxis behavior as a scale ranging -1.0 ≤ CI ≤ 1.0. For salt chemotaxis, CIs can show that a worm population is completely attracted to low salt (-1.0), displays a neutral response (+0.0), or is all attracted to high salt (1.0).*

24. Perform **steps E21–23** until all CTX plates have been used to calculate CIs for each CTX plate.

*Notes:*

*1. The CI for one CTX plate represents a measurement for one technical replicate of one group. Since three CTX plates are prepared in this protocol for each group, it is expected that you will have three technical replicate measurements for each group in one biological replicate, which can be averaged to indicate the overall behavior of the group.*

*2. Ideally, naïve and mock-trained animals will display high CIs (~0.7–0.9) and trained animals will output CIs that are between ~0.0 and 0.4 to represent successful salt associative learning by pairing “no salt” with “food” (* ***Figure 3B***).

*3. We generally accept biological variability between these groups for average CIs ± ~0.3, as long as trained animal behavior is statistically significant vs. negative learning groups in the same line. Behavioral experiments should be repeated in the instance that either naïve or mock-trained groups do not display high salt attraction (i.e., they show an average CI ≤ 0.6). This is also the case when trained animals behave similarly to naïve animals, in that they do not display the expected learned behavior (i.e., they present an average CI ≥ 0.6). See our Troubleshooting section for more information.*

25. CTX plates can be discarded after use on **day 13**.

26. Plot the average CI values to compare naïve, mock-trained, and trained cohorts for each strain. We used a two-way ANOVA to compare means (strain, group). The expectation is that a comparison between naïve (or mock-trained) vs. trained animals within strains should be statistically significant, to indicate that animals have notably changed salt chemotaxis behavior in a way that is specific to pairing food with no salt (**Figure 3B**). Non-significance would suggest a learning defect, meaning that animals cannot be used for downstream proteomics. In contrast, comparisons between naïve and mock-trained groups within strains should be non-significant (**Figure 3B**), as otherwise it indicates a general chemotaxis defect that limits the capacity to determine learning ability.

*Note: See Troubleshooting in the case that animals do not display the anticipated learned behavior after training, compared to naïve and mock-trained animals (Problem 1).*

**Pause point:** Subsequent steps involve protein extraction and processing steps from animals stored in a freezer (outlined in **step E17**). You can pause here since *C. elegans* pellets frozen at -80 °C can be used for downstream steps within 6 months.

**F. Extraction and quantification of total protein from whole *C. elegans***

Protein extraction methods detailed below are adapted from [38] and [25].

**(Day 14)**

1. Protein extraction: Set up a sonication system in a 2–8 °C room to run at 25% amplitude with a 4 s cycle (2 s on, 3 s off) per manufacturer instructions.

**Pause point:** Sonication runs can take several hours when samples are all processed on the same day.

2. Thaw *C. elegans* pellets in 1.5 mL centrifuge tubes on ice for 2 min, for one experimental group from one biological replicate.

*Note: It is expected to have three or four tubes with 50–100 μL of* C. elegans *per tube. Ideally, you will have a volume of packed worms that is greater than or equal to 150 μL for each group per replicate to extract the required amount of protein for downstream experiments. In our experience, a 150 μL packed pellet yields ~1 mg of total protein.*

3. Add 200 μL of u-RIPA to each thawed *C. elegans* pellet.

*Note: We use BCA assays to assess protein concentration by colorimetric assay [39]. As urea can break down into cyanate over time [40], and cyanate can function as an oxidizing agent through a reduction reaction to cyanide [41], it is possible that cyanate in older u-RIPA solutions may impact copper ions used in the assay and affect measurements made by BCA. u-RIPA should be prepared fresh on the day that sonications are performed on* C. elegans *to avoid this potential issue.*

4. Sequentially sonicate each *C. elegans* pellet for one group until each pellet has undergone 10 sonication runs at 25% amplitude and a 4-s total sonication time per sonication. Allow each pellet to recover on ice for >20 s in-between sonications to minimize sample degradation.

5. Repeat **steps F2–4** until all *C. elegans* pellets have undergone 10 sonication runs. Keep sonicated *C. elegans* samples on ice at all times until centrifugation (the next step in this section).

*Note: The probe must be cleaned between groups and replicates to minimize cross-contamination. This can be done by performing one sonication run with ultrapure water in a beaker and then wiping the sonicator probe with ethanol with a Kimwipe.*

6. Spin down sonicated *C. elegans* samples using a refrigerated centrifuge (14,000 rcf, 10 min, 4 °C). This centrifugation will result in each tube containing the following three layers from top-to-bottom: a translucent lipid layer, a clear protein layer, and a pellet of worm carcasses. Label new 1.5 mL tubes during the 10-min incubation with relevant sample information.

7. For each sample, transfer the clear protein layer from each spun-down 1.5 mL tube to its new labeled 1.5 mL tube. This is the total protein from whole bodies in a worm population, and it contains both non-biotinylated and biotinylated proteins.

**Pause point:** Total protein can be stored in an ultracold -80 °C freezer for use within one year for this experiment.

8. Quantification of total protein by BCA assay: Generate one 1:15 diluted aliquot for each sample by pipetting 1.6 μL of total protein and 22.4 μL of u-RIPA into a new 1.5 mL tube. Pipette 10 μL of each diluted aliquot into two separate wells in a 96-well plate, to run each sample in duplicate.

*Note: Total protein samples should be kept on ice during* ***steps F8–13*** *when setting up and performing the BCA assay. These samples will be used in* ***section G*** *for downstream proteomics.*

9. Perform a serial dilution with one vial of bovine serum albumin (BSA) (2 mg/mL) from the Pierce^TM^ BCA Protein Assay kit to generate eight 1.5 mL tubes with different known concentrations of BSA in u-RIPA as standards (see **Table 1** for details). Add BSA aliquots to the 96-well plate to run each standard in duplicate (10 μL per well).

*Note: Ensure that these standards are prepared fresh. The remaining BSA (2 mg/mL) can be stored long-term in a refrigerator (4 °C) and used for at least one year.*

**Table 1.** Volumes to make up fresh BSA standards for BCA. Each standard is named with one letter between A and I. All standards are diluted in u-RIPA for this section, or RIPA in **section G**. Numerical values represent volumes in μL.

BSA standard	BSA source for standard	Volume of diluent	Volume of BSA source
B	A (from the kit)	12.5	37.5
C	A (from the kit)	32.5	32.5
D	B	17.5	17.5
E	C	32.5	32.5
F	E	32.5	32.5
G	F	32.5	32.5
H	G	40.0	10.0
I	No source	40.0	0.0

This table is adapted from the user manual made by Thermo Fisher Scientific for the Pierce^TM^ BCA Protein Assay kit.

10. Follow the manufacturer’s instructions to perform the BCA assay.

11. Incubate the 96-well plate containing diluted protein aliquots at 37 °C for 15 min, then measure absorbance values at 562 nm using an appropriate spectrophotometer or imager.

12. After exporting values from the imager, calculate average absorbance values for standards and generate a 562 nm standard curve with a linear fit. Use the linear equation to estimate sample protein concentrations, correcting for the 1:15 dilution. Calculate the volume corresponding to 1 mg total protein and prepare aliquots accordingly.

13. Boil one aliquot containing 40 μg of total protein with 5× sample buffer (8 μL) and u-RIPA (up to 40 μL total volume) per sample at 95 °C for 3 min, and then cool samples on a tube rack to room temperature for ~10 min. These samples will be used to assess biotinylation levels in total protein caused by TurboID (in YLC207 animals) vs. signal from endogenously biotinylated proteins only (in N2 groups) by western blot (see General note 7 and Problem 3). They can be stored long-term at -80 °C.

**G. Biotinylated protein enrichment and digestion for LC–MS/MS**

This section involves (i) performing a buffer exchange for each total protein sample to reduce excess biotin and urea levels, to maximize (ii) enrichment of biotinylated proteins by pull-down using streptavidin. Protein samples then undergo (iii) reduction with DTT and alkylation using iodoacetamide, and (iv) trypsin-mediated protein digestion. All protocols for streptavidin-mediated pull-down, protein reduction and alkylation, and trypsinization of biotinylated proteins bound to streptavidin-coated magnetic beads are adapted from [30] and [26]. Peptides produced from the trypsin digest are processed in **section H** so that they can be run in a mass spectrometer.

**(Day 15)**

1. Buffer exchange with desalting columns: Remove seals from the bottom of each 7 kDa molecular weight cutoff desalting spin column and then place each column in an open 2.0 mL centrifuge tube with the column lid loosened (but not fully open).

*Notes:*

*1. These columns will be used to perform a buffer exchange with 1 mg of total protein from each experimental group according to the manufacturer’s instructions [25].*

*2. The buffer exchange reduces excess biotin from the biotin treatment on* ***day 12***, *as excess biotin will interfere with pull-down attempts for biotinylated proteins from total protein with streptavidin-coated magnetic beads.*

*3. Urea can weakly bind to streptavidin [42], so the buffer exchange also serves to reduce urea levels from u-RIPA to maximize capacity for streptavidin to bind biotinylated proteins only.*

*4. Store total protein aliquots on ice during* ***steps G1–13***.

2. Centrifuge each column in an open 2.0 mL tube (1,500 rcf, 1 min, 4 °C) and then discard the elution.

3. Use a marker pen on each column to record the direction that the resin slanted toward following the centrifugation run in **step G2.** All subsequent centrifugation runs with these columns should be done so that the mark is facing outward from the centrifuge.

4. Rinse the resin in each column into the same 2.0 mL tube as in **step G2** with 300 μL of RIPA by centrifugation (1,500 rcf, 1 min, 4 °C) three times, discarding the solution in each 2.0 mL tube after each centrifugation run.

5. Centrifuge 30–130 μL of total protein for elution in RIPA using one column and a new 2.0 mL collection tube (1,500 rcf, 2 min, 4 °C).

*Note: Before centrifugation, add a further 15 μL of RIPA to each column with less than 70 μL of total protein.*

6. If 1 mg of total protein exceeds 130 μL, process the sample in two runs through the desalting column. Reuse the same column once by repeating **steps G4–5** to elute an additional 30–130 μL into the same tube. As columns can be reused only once, if the total volume exceeds 260 μL, repeat the full procedure with a new column.

7. Quantification of desalted total protein by BCA assay: Perform a BCA as in **steps F8–12**, but use BSA standards diluted in RIPA instead of u-RIPA. You will need 0.6–1.0 mg of desalted protein to proceed with subsequent steps.

**Pause point:** Desalted protein can be stored in an ultracold freezer for use within one year.

**Critical:** The steps below are time-intensive and have no pause points until **day 17**, so plan accordingly.

See Troubleshooting in the case that an insufficient amount of protein is detected from samples of interest (Problem 2).

9. Pull-down for biotinylated proteins, using streptavidin: Transfer four 394 μL aliquots of streptavidin-coated magnetic bead slurry into separate 2.0 mL tubes using an unfiltered pipette tip (1,000 μL, with ~0.5 cm of its tip end cut off).

10. Add 1 mL of TBST to each bead slurry aliquot, briefly spin down beads using a benchtop minicentrifuge for 3 s, and then incubate bead slurry aliquots on a magnetic rack on ice for ~2 min (until solutions are clear, instead of brown). Replace the supernatant with 1 mL of TBST, and then gently mix beads by spinning each tube while it sits in the magnetic rack until the solution is homogenously brown. This is one “quick wash.”

11. Equilibrate bead slurry aliquots by performing two more quick washes as in **step G10**.

12. Replace supernatant for each bead slurry aliquot with 0.6–1.0 mg of desalted protein and then add TBST until the bead slurry is in 1 mL of total volume.

13. Use the magnetic rack to gently mix desalted protein and streptavidin-coated beads in TBST, as in **step G10**, and then transfer 2.0 mL tubes onto ice until mixing is completed for protein from all four groups.

14. Incubate 2.0 mL tubes containing desalted protein in bead slurry in TBST on an appropriate laboratory mixer. We used the ELMI Intelli-Mixer^TM^ RM-2M (mode F2, 6 rpm) in a cold room (2–8 °C) for 18 h. This step serves as the streptavidin-mediated pull-down to encourage binding of biotinylated proteins to streptavidin-coated beads, since streptavidin has a high binding affinity to biotin [43].

**(Day 16)**

15. Briefly spin down 2.0 mL tubes containing total protein in bead slurry in TBST using a benchtop minicentrifuge for 3 s and then incubate 2.0 mL tubes on a magnetic rack on ice for ~3 min (until solutions are clear, instead of brown).

16. Transfer the supernatant to a new labeled 1.5 mL centrifuge tube. This solution contains proteins that have not bound to the streptavidin magnetic beads (i.e., non-biotinylated proteins). Following supernatant removal, immediately add 1 mL of TBST to the beads containing biotinylated proteins enriched from desalted total protein.

*Note: Keep 1.5 mL tubes containing supernatant from streptavidin-mediated pull-down on ice on* ***day 16***. *These samples will be used in* ***step G28***.

17. Gently mix biotinylated proteins bound to beads in solution as in **step G13**, and then incubate biotinylated proteins in bead slurry on the ELMI Intelli-Mixer^TM^ (mode F2, 6 rpm) or similar in a cold room (2–8 °C) for 5–10 min.

18. Use a benchtop minicentrifuge to briefly spin down 2.0 mL tubes containing enriched biotinylated proteins for 3 s.

19. Allow magnetic beads to separate from the solution. Place 2.0 mL tubes with biotinylated proteins on a magnetic rack on ice for ~3 min (until solutions are clear, instead of brown).

20. Replace supernatant in each 2.0 mL tube on the magnetic rack with 1 mL of TBST, discarding the supernatant.

21. **Steps G17–20** constitute a “thorough wash” of biotinylated proteins bound to streptavidin-coated magnetic beads. Repeat these steps once.

22. Spin down beads as in **step G18** and then aliquot 105 μL of bead slurry to new 1.5 mL tubes. These aliquots contain 60–100 μg of enriched biotinylated protein and will be used to assess the effectiveness of the streptavidin-mediated pull-down via western blotting.

*Note: These aliquots can be stored on ice during* ***day 16*** *until* ***step G29***.

23. Repeat **step G19** and then replace the supernatant in each 2.0 mL tube with 1 mL of 1 M KCl.

24. Perform six more thorough washes for enriched biotinylated proteins in each 2.0 mL tube as described in **steps G17–20**, but sequentially replace the supernatant with the following solutions: 0.1 M Na_2_CO_3_ (1×) and then PBS (5×).

*Note: All enriched biotinylated protein samples therefore undergo 10 thorough washes in total, using solutions in the following order in* ***steps G15–24***: *TBST (3×), 1 M KCl (1×), 0.1 M Na_2_CO_3_ (1×), and PBS (5×).*

25. Reduction: Replace PBS with 200 μL of PR solution for each 2.0 mL tube containing enriched biotinylated proteins.

26. Mix the PR solution into enriched biotinylated protein samples by (i) vortexing each 2.0 mL tube for 3 s and then (ii) briefly spinning down samples using a benchtop minicentrifuge for 3 s.

27. Allow PR solution to reduce disulfide bonds in biotinylated proteins bound to magnetic beads, by incubating 2.0 mL tubes on a thermomixer set to 55 °C and 800 rpm for 1 h.

*Note: You can proceed with* ***step G28*** *during this 1-h incubation.*

28. Preparing proteins for western blot to assess pull-down: For the experimental group and replicates, prepare one 1.5 mL tube with 8 μL of 5× sample buffer and 32 μL of supernatant preserved in **step G16**.

29. Separate beads from supernatant for 105-μL bead slurry aliquots (from **step G22**) and replace the supernatant with 40 μL of 1× sample buffer per tube.

30. Briefly vortex 1.5 mL tubes containing proteins in sample buffer for 3 s and then boil these samples in a thermomixer (0 rpm, 95 °C, 3 min). Allow boiled protein samples to cool to room temperature for at least 5 min before storage in an ultracold freezer for use within one year. These samples will be used to evaluate the efficiency of enrichment for biotinylated proteins by western blotting (see General note 7).

*Note: See Troubleshooting if biotinylated proteins cannot be seen in a western blot (Problem 4).*

31. Alkylation: Add 4 μL of 0.5 M iodoacetamide to each 2.0 mL tube containing biotinylated proteins in PR solution.

32. Repeat **step G26** to mix iodoacetamide and then allow iodoacetamide to alkylate broken disulfide bonds within biotinylated proteins by incubation in a thermomixer (800 rpm, 55 °C, 20 min).

*Note: Iodoacetamide is light-sensitive [44], so limit the amount of light that samples are exposed to during this incubation.*

33. Digest with trypsin: Add 162 μL of digestion solution containing trypsin to each 2.0 mL tube containing biotinylated protein that has undergone sequential reduction and alkylation reactions.

34. Repeat **step G26** to mix proteins with the digestion solution and then incubate the corresponding 2.0 mL tubes in a thermomixer (800 rpm, 37 °C, 18 h) to promote trypsin digestion of proteins into peptides.

**(Day 17)**

TurboID biotinylates lysine residues in nearby proteins [19]. Trypsin-mediated digestion of proteins labeled with this enzyme will therefore result in a mixture of non-biotinylated peptides and biotinylated peptides. The digestion reaction by trypsin is stopped/quenched using TFA (in **steps G38–41**). On **day 16**, trypsin was introduced to proteins bound to streptavidin-coated magnetic beads, meaning some peptides will be bound to the beads due to ≥1 biotin residues, and hence require elution before TFA (**steps G42–48**). Remaining peptides lack a biotin label and are referred to as “unbound” peptides in this protocol. These peptides are immediately treated with TFA.

35. Use a benchtop minicentrifuge to briefly spin down each 2.0 mL tube for 3 s, following the 18-h incubation with trypsin (from **step G34**). Use a magnetic rack to separate beads from the solution at room temperature until the solution is clear, instead of brown; this typically takes less than 2 min.

**Critical:** All steps that mention pipetting on **day 17** must be performed in an appropriate fume hood, since solutions contain hazardous chemicals.

36. Transfer the supernatant from each tube on the magnetic rack into a new 2.0 mL tube.

*Note: This solution contains non-biotinylated peptides that are not bound to streptavidin (i.e., unbound samples). These peptides will undergo the TFA treatment in* ***steps G38–41***.

37. Immediately add 250 μL of EB solution to each 2.0 mL tube containing magnetic beads to begin the elution process for biotinylated peptides bound to streptavidin.

*Note: Peptides in bound samples will be eluted from beads (* ***steps G42–48*** *), and then the eluted peptides will undergo TFA treatment (* ***steps G38–41***).

38. First TFA treatment: Centrifuge 2.0 mL tubes containing unbound samples (20,000 rcf, 22 °C, 20 min), following the addition of 4 μL of 10% (w/v) TFA to each tube. This is done to remove solid debris from peptide solutions (e.g., streptavidin-coated magnetic beads).

*Notes:*

*1. You can proceed with* ***steps G42–48*** *during the centrifugation runs in* ***steps G38 and G41***.

*2. Store peptide samples for* ***days 17–19*** *on ice if they cannot be processed within 30 min, to minimize contamination during short-term storage.*

39. Pipette the clear solution in the middle of each centrifuged tube containing unbound peptides into a new 1.5 mL tube.

40. Second TFA treatment: Add 400 μL of 0.1% (w/v) TFA to each tube containing unbound peptides, and then vortex unbound peptides in 0.1% (w/v) TFA for 3 s.

41. Perform a centrifugation run (20,000 rcf, 22 °C, 10 min) with unbound peptides in 0.1% (w/v) TFA and then add the clear solution in the middle of each spun-down 2.0 mL tube to its corresponding 1.5 mL tube from **step G39**.

*Note: These 1.5 mL tubes contain non-biotinylated peptides that will be desalted with tC18 cartridges, vacuum-concentrated, and then resuspended in an LC–MS/MS-compatible buffer for mass spectrometry runs (see* ***section H***).

42. First elution of bound peptides: There are two elution steps to maximize peptide recovery. For bead slurry containing bound peptide samples in EB solution, from **step G37**, mix until the solution is homogenously brown by alternating use of a magnetic rack (to spin tubes on the rack) and vortex (in 3-s bursts).

43. Incubate samples in a mixer, e.g., ELMI Intelli-Mixer^TM^ (mode F2, 6 rpm), for 5 min at room temperature.

44. Use a magnetic rack to separate beads from solution in 2.0 mL tubes containing bound peptide samples (see **step G35**) and then transfer the supernatant containing eluted biotinylated peptides to a new labeled 1.5 mL tube for each sample.

45. Second elution of bound peptides: Add 200 μL of EB solution to the original 2.0 mL tubes containing bead slurry and bound peptide samples.

46. Mix bound peptides in 200 μL of EB solution by repeating **step G42**.

47. Elute any remaining bound biotinylated peptides from streptavidin-coated magnetic beads by incubating tubes containing beads and bound peptides in EB solution on a temperature-controlled mixer (800 rpm, 95 °C, 5 min).

**Critical:** Tube lids may open during this step due to pressure buildup, risking exposure to hazardous reagents and sample loss. To prevent this, secure tube lids with the mixer lid and sticky tape, hold the lid down during the 5-min incubation, and carefully remove tubes using the rack cover while keeping lids closed.

48. Repeat **step G35**, then transfer the supernatant containing additional eluted biotinylated peptides from each bound sample into the corresponding 1.5 mL tube from **step G44**.

49. Use TFA to treat combined elutants for bound samples by repeating **steps G38–41** to quench any remaining trypsin.

**Pause point:** All TFA-treated peptides (from **steps G41 and G49**) can be stored in a -80 °C freezer for use within two weeks.

**H. Preparing peptides digested from biotinylated proteins for LC–MS/MS**

This section is adapted from [30].

**(Day 18)**

1. Desalting peptides with tC18 cartridges: Allow TFA-treated peptide samples from -80 °C to thaw on ice for 1 h, by lying each 1.5 mL centrifuge tube on its side on top of the ice.

**Critical: Steps H2–7** must be performed in a fume hood, as you will be handling solutions containing hazardous chemicals.

*Note: Solutions used to desalt peptides can be stored at room temperature in an appropriate flammables cabinet and used for this experiment within one year.*

2. For each peptide sample, (i) remove the plunger from one 3 mL syringe, (ii) insert the larger opening of one tC18 cartridge onto the syringe, (iii) add 3 mL of CE solution into the syringe with a manual pipette (100–1,000 μL), (iv) insert the plunger into the syringe to eject CE solution into an appropriate waste container, and then (v) set aside the syringe and cartridge pair with the plunger still inside the syringe. Repeat this step for each pair of syringe and cartridge, so that you have one pair for each peptide sample.

*Notes:*

*1. Treat unbound and bound peptide samples from the same experimental group as different samples, for the purpose of determining how many syringes and cartridges you will need for this step. This protocol requires 8 pairs of syringes and tC18 cartridges for each biological replicate of peptide samples.*

*2. When dispensing from a syringe–cartridge pair, push the plunger with your thumbs while securing the cartridge against the syringe with your index fingers to prevent it from slipping under pressure.*

*3. Syringe-cartridge pairs can be reused within 5 years by repeating* ***steps H2–3***, *after* ***step G7.*** *They can be kept at room temperature in an airtight box for long-term storage.*

3. Repeat **step H2** with 3 mL of 50% (v/v) methanol per syringe-cartridge pair, so cartridges are clean/ready-to-use afterward.

4. Wash each syringe-cartridge pair three times by sequentially using the following solutions once as in **step H2**: (i) 3 mL of 100% (v/v) acetonitrile, (ii) 2 mL of CW buffer, and (iii) 3 mL of 0.1% (w/v) TFA.

5. Load all ~400 μL of TFA-treated peptide sample into one syringe-cartridge pair and then dispense the solution in a waste container as in **step H2**. Peptides should stay in the cartridge; this will be validated later.

*Note: Ensure that you keep track of which sample was used in which syringe-cartridge pair.*

6. Sequentially wash each syringe-cartridge pair with (i) 2 mL of 0.1% (w/v) TFA and then (ii) 200 μL of 0.5% (v/v) acetic acid in ultrapure water as in **step H2**.

7. Carefully eject 800 μL of CE solution from each syringe-cartridge pair into a new labeled 1.5 mL tube; this contains peptides desalted to remove excess TFA.

8. Preparing peptide samples in an LC–MS/MS-compatible solution: Use a vacuum concentrator as per the manufacturer’s instructions to remove solutions from each desalted peptide sample and retain peptides that will be visible as dried powder. This step will take approximately 2.5–5.5 h.

*Note: Peptides are suspended in mostly acetonitrile, so open the ballast during vacuum concentration runs.*

**Pause point:** These peptide samples can be stored in an ultracold freezer for use within two weeks.

**(Day 19)**

9. In an operating biosafety cabinet, thoroughly coat the walls of each 1.5 mL tube containing dried desalted peptides with 35 μL of MSRB solution to resuspend peptides, and then transfer each peptide sample into LC–MS/MS-compatible vials.

10. Briefly spin down 1.5 mL tubes containing peptides in MSRB using a minicentrifuge for 3 s at room temperature.

11. Determine the peptide concentration in each sample resuspended in MSRB. We used the “1 Abs = 1 mg/mL” setting on the Nanodrop One according to the manufacturer's instructions (see Troubleshooting, Problem 5).

*Notes:*

*1. Peptides can be transferred to LC–MS/MS-compatible vials after this step.*

*2. We usually read ~2.0 μg/μL for bound peptide samples and ~0.2 μg/μL for unbound peptide samples.*

**(Day 20)**

12. Perform LC–MS/MS runs with suitable machines (3 μg per sample), according to the manufacturer’s instructions.

*Note: We highly recommend that users first perform a pilot experiment to optimize acquisition parameters before larger-scale experiments. For example, you can run peptides from two naïve groups as your pilot: (1)* C. elegans *with no TurboID vs. (2) TurboID-expressing worms.*

See **Troubleshooting, Problem 6**, if LC–MS/MS runs fail to detect a sufficient amount of protein identities corresponding to *C. elegans*.

**I. Analyzing proteomic data using the MASCOT database**

Mass spectrometry can enable comparisons of proteins expressed between experimental groups. Our goal was to identify neuronal proteins present only in the learning proteome, and not in the mock-trained proteome. This is why we utilized a simple subtraction method for qualitative analysis (detailed below).

**(Day 21)**

1. Upload each raw LC–MS/MS file (in .raw file format) to the MASCOT search engine, using the *MS/MS ion search* option with an appropriate *C. elegans* Swiss-Prot database and the following settings: *semi-trypsin* enzyme, *monoisotopic* mass values, *unrestricted* protein mass, *±5 ppm* peptide tolerance, *±0.05 Da* fragment mass tolerance, and *3* maximum missed cleavages. Select *Biotin (K), Carbamidomethyl (C), Oxidation (M), Phospho (ST)*, and *Phospho (Y)* as variable modifications.

*Note: The Matrix Science website includes tutorials that are readily available under the* Help *tab for MASCOT search engine use:* *https://www.matrixscience.com/help.html* .

2. The MASCOT search engine will output a report in either the “peptide summary” or “protein family summary” format. Navigate to the drop-down menu next to the *Format as* button in the user interface of MASCOT, in your report. Select the *Peptide summary* option and then *Format as* to change the report from “protein family summary” to “peptide summary.”

*Note: See Troubleshooting if an insufficient amount of protein identities is detected by MASCOT (Problem 6) and if you are unable to generate peptide summary reports (Problem 7).*

3. Repeat **step I2** but select the *Export search results* option to export your report from MASCOT as a .csv file.

4. Perform **steps I1–3** for all raw LC–MS/MS files.

*Note: Ensure that each file is appropriately labeled with the biological replicate number, genotype (non-transgenic vs. TurboID), experimental group (mock-trained vs. trained), and sample type (bound vs. unbound). These files contain information for peptides* assigned *a protein identity by MASCOT (we refer to these as “assigned hits”).*

5. On the STRING website, navigate to the *multiple proteins* search option and then paste the list under the *prot_acc* column from one .csv file into the search box (from **step I3**). Select the organism *Caenorhabditis elegans* on STRING and then click *Search*.

6. Paste the table under *Your input* in the *Legend* tab of the STRING webpage into a new spreadsheet. There are two columns: (i) gene names and (ii) descriptions for known or predicted gene functions. The leftmost column will contain gene names for assigned hits from one sample; save this information as a new file.

7. Repeat **steps I5–6** for all .csv files exported from MASCOT, to convert protein identities into gene names for all samples run via LC–MS/MS. Copy each table in a new tab on the spreadsheet from **step I6**.

8. For the spreadsheet from **step I6**, in a new tab, combine gene name lists from bound and unbound sample types (from **step I7**) for those that share the same biological replicate, genotype, and experimental group. To do this, paste both lists in the same column and then use the function *Data* → *Remove duplicates* in Microsoft Excel (or equivalent software).

9. Identify proteins present in *C. elegans* neurons during memory encoding for each biological replicate (i.e., in gene name lists for trained animals expressing TurboID), by subtracting gene names also detected from animals in control groups from the same replicate. In our experiments, each control group contains animals that did not express a TurboID enzyme [trained + no TurboID (N2 line)] and/or were not trained by salt associative learning [mock-trained + TurboID (YLC207 line)].

10. Combine replicate-specific gene name lists unique to trained animals with TurboID as in **step I8**, with lists output by **step I9**. This will generate a putative list of candidate learning regulators from this experiment as a “learning proteome” of assigned hits from the whole *C. elegans* nervous system.

**J. Annotation of “unassigned hits” using BLAST and Python**

This section uses a custom Python code that automates bulk searching for peptide sequences (peptide score ≥ 15) without a unique peptide, and therefore not assigned a protein identity by the search engine MASCOT. Searching is done by this code using the *Reference proteins (refseq_protein)* database and *Caenorhabditis elegans (taxid:6239)* organism option. It outputs protein identities when one (or more) peptide sequences share a 100% identity match with the protein and an e-value < 0.05, where a smaller e-value represents an increased probability that the identity is true and not given by random chance. Problem 8 is provided below in case the software mentioned in this section does not function as intended.

**(Day 21)**

1. When a peptide summary report is opened as a webpage in your preferred browser, right-click on the webpage to select *Save as* and then save the report as an .html file using the *webpage, complete* file type option.

*Note: Peptide summary reports can be of substantial file size, requiring significant time to load depending on your computer setup, so handle one report at a time to minimize lag during this step.*

2. Save each .html file in its own folder within one master folder and then copy the newest versions of all GitHub files for our custom Python code to each sample’s folder (Chew Worm Lab, 2025, *Python code for BLASTp search of undetected C. elegans hits;* see **Software and datasets** for the GitHub URL).

3. Open the VS code program, select *Open folder* in the *Welcome* screen/tab, and then open the master folder containing all .html files. Click *>* to the left of a subfolder name to visualize its contents.

4. Open a single .html file in VS Code and use *Ctrl* + *F* to locate Peptide matches not assigned to protein hits: to access the section containing unassigned peptides.

5. Place the cursor at the beginning of the first instance of <tr><td align="RIGHT"> in the paragraph of the .html with unassigned hit information, by clicking onto the left side of the first character in the same line.

6. Scroll through the .html file in VS code until you reach the end of the paragraph containing unassigned hit information, marked with the line </tbody></table>. Select the entire body of text.

7. Copy the text from the MASCOT .html and then paste the information into the htmlTemplate.html file from GitHub. During this step, paste the paragraph so that it replaces ##paste table here (delete this line) in htmlTemplate.html. Save the changes made to this file by simultaneously pressing buttons *CTRL* + *S* on your keyboard.

8. In VS code, press *CTRL* + *Shift* + *P* to open a pop-up menu, select the option *Python: Select Interpreter*, and then select *base (3.9.12)*.

9. Update line 9 of “main.py” from GitHub with the new file path for htmlTemplate.html within quotation marks (e.g., ”peptideFile=pathlib.PureWindowsPath(r“C:\LCMSMS\Replicate_1\N2_trained_unbound\htmlTemplate.html”).

10. Navigate through the top-menu options *Terminal* → *New terminal* to open a new command prompt in VS code (marked as “cmd”). This should automatically convert to an Anaconda environment in the terminal window, labeled with (base).

*Note: You can also type the file path for the activate.bat file from the Anaconda 3 installation files, downloaded to the Program files folder by default (e.g., C:\Program Files\Anaconda3\Scripts\activate.bat). This will force an Anaconda environment to open in the terminal.*

11. You may need to manually navigate to main.py to run the Python code in Anaconda through VS code. To do this, (i) to switch to the correct drive containing main.py, type the letter of the drive with a colon in the terminal and then press *Enter* (e.g., C:), (ii) enter cd (change directory) and the file path for main.py in the terminal (cd C:\LC–MS/MS\Replicate_1\N2_trained_unbound), and then (iii) run the Python code main.py by entering python main.py into the Anaconda environment.

*Notes:*

*1. This will generate an* Overview of Peptide Processing.txt *file. This file can be used to check that the expected .html file has been processed, by comparing the first biotinylated query number in this .txt file versus the edited* htmlTemplate.html. *Note that the first biotinylated query number will be for the first peptide listed with the* Biotin (K) *modification.*

*2. .txt files annotated with* blastSeq *in their file name contain lists for peptide sequences reformatted from* htmlTemplate.html *to be compatible for upload onto BLAST for bulk searching.*

*3.* BLAST meta *.txt files are produced by the Python code to inform the version of BLAST used for bulk searching.*

*4.* Protein-Hits *.csv files contain the results from bulk BLAST searching, converting peptide sequences into protein identities, as well as a record of instances that the protein was identified from the original unassigned peptide data.*

*5. .txt files annotated with* gene_Ids *in their file names contain protein identities from peptide sequences. These identities are in the form of protein accession numbers, which will be converted to gene names in subsequent steps.*

*6. Each file will be labeled as “biotin” for peptide sequences listed with the* Biotin (K) *modifier or “no biotin” for those without the* Biotin (K) *modifier. Note that both types of peptides would have been digested from biotinylated (neuronal) proteins by trypsin, given that the proteins were bound onto streptavidin-coated magnetic beads.*

12. Repeat **steps J4–11** until all .html files from the MASCOT database have been analyzed.

13. On the Batch Entrez^®^ website (see **Software and datasets**), select the gene database and upload one of the .txt files output from the custom Python code that is annotated with *gene_Ids*. Click *Retrieve* and then the retrieve records hyperlink. Choose the *File* destination and *Tabular (text)* format under the *Send to* drop-down menu from the results webpage on Batch Entrez^®^, to save the results. The *Name/Gene ID* column contains gene names converted from protein accession numbers in the uploaded *gene_Ids* .txt file. Repeat this step for all *gene_Ids* files.

14. Repeat **steps I8–10** to identify unassigned hits uniquely present in *C. elegans* neurons during memory encoding of salt associative learning.

**Figure 4.**
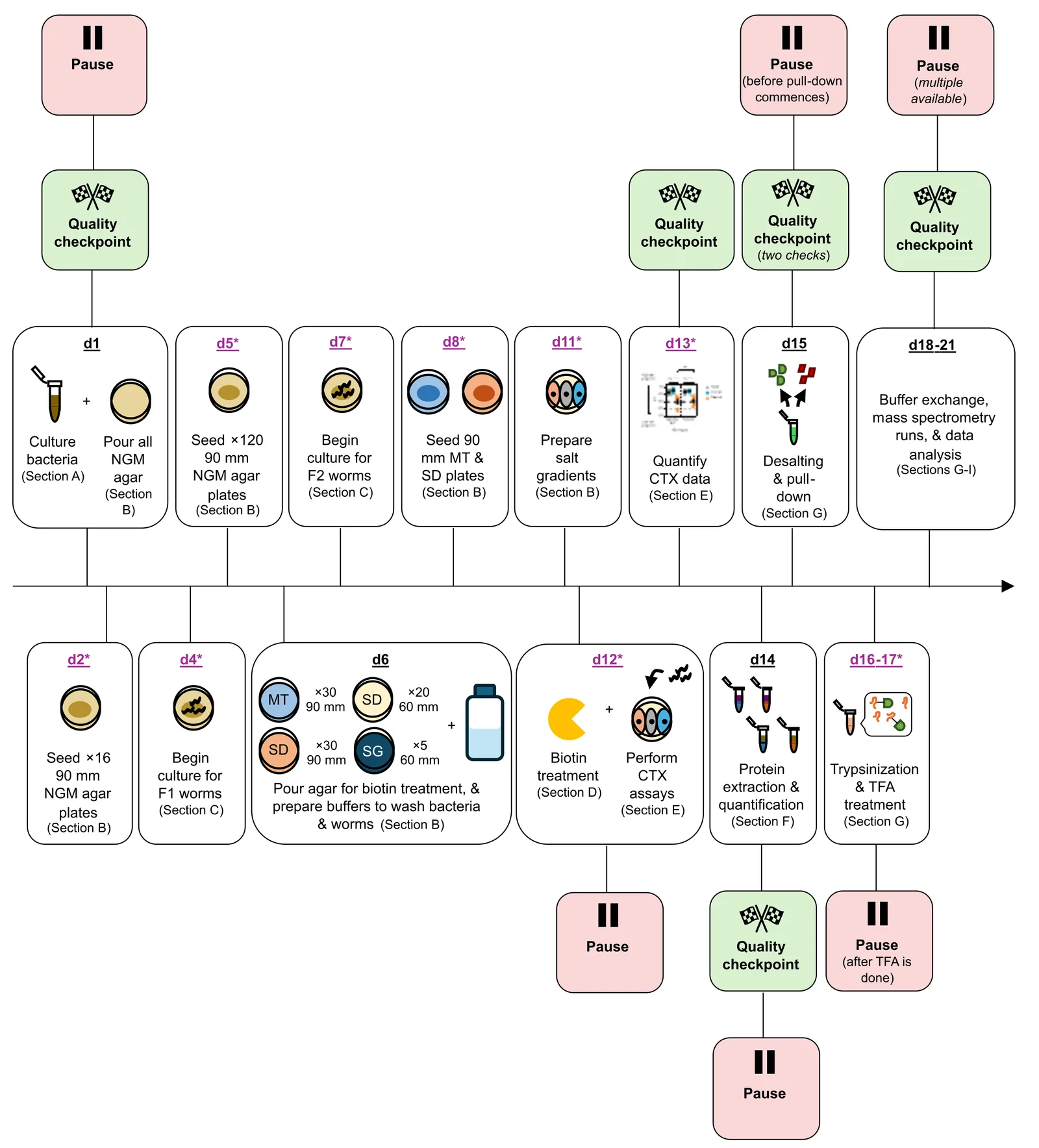
Timeline of the workflow, including pause points and quality-control checkpoints. This protocol can be completed in approximately three weeks and includes eight pause points (including multiple pause points within **days 18–21**, i.e., after TFA treatment) to improve flexibility. Bacterial cultures, NGM plates, and washing buffers can be prepared up to three weeks in advance. Worm pellets collected after **day 12** may be stored at -80 °C for up to one year, while extracted and desalted protein samples (**days 14–15**) may be stored for up to six months. Following TFA treatment on **day 17**, peptides may be stored at -80 °C for up to two weeks between buffer exchange, resuspension in LC–MS/MS buffer, and mass spectrometry analysis steps. Data analysis can be performed at any time following data acquisition. **Days 2–13** and **days 15–17** (in purple and marked with an asterisk) should be performed according to the prescribed schedule. Consistency across **days 2–13** is important because learning and behavior are highly sensitive to experimental conditions. In addition, immobilized worms should be counted immediately following behavioral assays on **day 13**, as carcasses may deteriorate during prolonged storage. Protein enrichment and trypsin digestion (**days 15–17**) should be completed without interruption to minimize freeze/thaw cycles and maximize peptide recovery. Six quality-control checkpoints are incorporated throughout the workflow. These include (1) assessment of bacterial culture growth (**day 1**), (2) behavioral performance by chemotaxis assay (**day 13**), (3–4) protein quantification in two separate experiments (i.e., before and after desalting on **days 14 and 15**, respectively), (5) verification of protein biotinylation and streptavidin-mediated enrichment by western blotting (**day 15**), and (6) peptide quantification prior to LC–MS/MS analysis (**day 19**).

*Note: All sections in this procedure are summarized in* ***Figure 4***.

## Data analysis

**Chemotaxis assays**: An ordinary two-way ANOVA with Tukey’s multiple comparisons post-test (α = 0.05) was done in GraphPad Prism to compare mean CI values between each experimental group. Biological replicates were excluded when bacterial contamination was evident, or fewer than 20 animals were present in a technical replicate. Eight biological replicates were initially done to test animals for learning, with three technical replicates per biological replicate. Three biological replicates were pilot experiments aiming to assess behavior only, whereas the remaining five involved more animals and were used for downstream proteomic experiments (n ≥ 3,000 animals per experimental group). Expertise using GraphPad Prism or similar statistics software is required for this analysis. More details are available in [14].

**LC–MS/MS**: In our experiments, five biological replicates were performed using mass spectrometers Q-Exactive Orbitrap and/or Orbitrap Exploris. We recommend three biological replicates at a minimum, in line with previous works that use proximity labeling in *C. elegans* [25–26]. It is optional to run technical replicates. Peptide samples from one biological replicate were run on both machines, meaning that six lists of proteins unique to TurboID-trained animals constitute the learning proteome data identified in [14]. This information is summarized from [14].

**Computational data analyses**: The following settings were used to assess our LC–MS/MS data using the **MASCOT search engine**: *semi-trypsin* enzyme specificity, *monoisotopic* mass values, *unrestricted* protein mass, *±5 ppm* peptide tolerance, *±0.05 Da* fragment mass tolerance, and *3* maximum missed cleavages. *Biotin (K), Carbamidomethyl (C), Oxidation (M), Phospho (ST)*, and *Phospho (Y)* were selected as variable modifications in MASCOT. In the instance that further quality control is necessary for your data, you can implement stricter criteria (e.g., peptide score(s) ≥ 40 and protein score(s) ≥ 30, as in [29]). We filter peptide information based on three variables for **bulk BLAST analyses with Python**: (1) peptide score ≥ 15 calculated by MASCOT, (2) a 100% identity match between peptide sequences with a peptide score ≥ 15 and the protein determined by BLAST, and (3) an e-value < 0.05 for the identity match calculated by BLAST.

## Validation of protocol

This protocol has been used and validated in the following research article [open access]:

• Rahmani et al. [14]. Identifying regulators of associative learning using a protein-labelling approach in *Caenorhabditis elegans. eLife*. https://doi.org/10.7554/elife.108438

The following figures in our eLife paper validate (1) our learning paradigm by chemotaxis assay, (2) TurboID enzyme function via western blots, and (3) our proteome data by in-silico analyses:

1. **Figure 1B **: A chemotaxis assay graph that confirms animals can learn during proximity labeling. Note that this data is also shown as **Figure 3B** in this protocol.

2. **Figure 1C, Figure 1, figure supplement 1, and Figure 1, figure supplement 3**: Western blots that show TurboID can label proteins when biotin is administered to worms in the same environment and duration as training.

3. **Figure 2 **: A schematic that highlights that our proteome data includes known learning regulators when proximity labeling is targeted to the training phase of salt associative learning. This figure was generated by manual literature searching for studies that aimed to characterize learning in the worm. This was a crucial check because we used TurboID while animals were trained to learn, with the purpose of elucidating novel regulators for this process.

4. **Table 1**: This uses an established *C. elegans* transcriptome database to confirm that our proteome dataset includes many molecules known to be expressed in neurons. This is important since the TurboID enzyme was used to label proteins in neurons only.

## General notes and troubleshooting

**General notes**

1. Fungal and bacterial contamination influence egg-laying [45] and/or lifespan [46] (reviewed by [47]). In addition, gene expression is affected by infection from fungi or bacterial contamination (reviewed by [48]). Their presence during development, memory encoding (mock-training/training), and/or memory retrieval (chemotaxis assay) will therefore likely confound data output from behavioral experiments and mass spectrometry runs.

2. Once agar has been poured into Petri dishes or bacteria have been seeded onto agar, the biosafety cabinet can be left non-operational while the agar or bacteria solidify overnight in a clean environment. This requires the cabinet door to be closed and plate lids to be left on their Petri dishes during this period.

3. The chapter “Maintenance of *C. elegans*” in WormBook [31] has a detailed overview of key steps, including preparation of a worm pick and methods to transfer *C. elegans* between plates. We recommend that users of this protocol who are less familiar with *C. elegans* refer to this chapter.

4. This protocol uses transgenic animals from line YLC207 that have been confirmed to express a functioning TurboID enzyme [14] vs. known wild-type (and non-transgenic) line N2. In the instance that you need to generate your own TurboID transgenic line, we recommend reading [26] for necessary experimental design considerations.

5. *C. elegans* are constantly swimming when in solution; it is important that animals are handled in solution quickly and consistently between experimental groups. Consistency is important so that each group has similar energy levels to each other [49], to reduce confounding factors that may influence behavior. Separately, speed is required since gustatory behavior can be modulated by exposure to salt in solution (with no food) for 15 min [13]. It is crucial that behavioral changes induced by changes in gustatory cues and food availability are induced only by associative learning encoded during biotin treatment, so that TurboID can capture the proteome changes from memory formation.

6. *Wicking* can be done with a Kimwipe folded three times and then rolled into a tight roll, by gently but quickly pressing an edge of the Kimwipe roll onto each droplet. Wicking needs to be done gently enough not to rip the agar, as animals can burrow into agar [50], affecting the sample size for biotin treatment. In addition, pressure from the wicking process should also not be applied such that it tears animal bodies, as the inclusion of animal corpses generated before biotin treatment will confound protein expression profiles output by mass spectrometry runs. This wicking process also needs to be done efficiently, given that moving too slowly will cause animals to be lost in the folds of the rolled Kimwipe, instead of remaining on agar for biotin treatment. Aliquots should be wicked according to how long animals have been in solution, prioritizing those aliquoted first until they have all undergone wicking.

7. All western blots were done by using standard methods [51].

**Troubleshooting**

**Problem 1:** Animals do not perform the expected behavioral change induced by salt associative learning (i.e., the suppression of naïve attraction toward high salt).

Possible causes:

1) Reagents may contain residual amounts of sodium chloride that impact the capacity to pair no salt + food to reduce naïve high salt attraction behaviors.

2) There may be bacterial or fungal contamination on your plates. This is likely from the bacteria or the agar itself.

3) The *C. elegans* line may have a background mutation that negatively impacts learning ability.

4) In the instance that you are using an integrated transgenic line, the gene for your TurboID enzyme may have integrated into a deleterious region in the worm genome.

Solutions:

1) Ensure that all plasticware and glassware used to generate agar plates and buffers (NSB and WWB) are, where possible, sterilized by autoclaving before use.

2) *E. coli* can be normally frozen in glycerol in an ultracold freezer for long-term storage. Use freshly rethawed *E. coli* from your glycerol stock. Remake NGM and LB plates fresh. Adult gravid worms can be treated with sodium hypochlorite (spot bleaching) to remove contamination, as detailed in the chapter “Maintenance of *C. elegans*” in WormBook [31].

3) For lines that initially behave as expected but then change unexpectedly, they likely obtained a spontaneous mutation, as this is possible in laboratory conditions [52]. Ultracold freezing is also viable as a long-term storage option for *C. elegans*. Therefore, you can retrieve fresh animals from these stocks, decontaminate them with bleach (as above), and then retry behavioral experiments.

4) Backcross the integrated transgenic line with wild-type animals at least four times to minimize background mutations. Animals that still do not perform the anticipated learning behavior likely have their integrated gene in a deleterious region, and therefore, another line needs to be made for this purpose. Refer to the protocol by [26] when planning to engineer a new line.

**Problem 2:** Insufficient amount of protein to proceed with downstream proteomics.

Possible causes:

1) Excessive worm loss during transfers between Petri dishes in-solution.

2) Incomplete worm lysis during sonication runs.

3) Protein loss during desalting.

Solutions:

1) Be as thorough as possible during washing steps, noting timing and logistical limitations.

2) Adjust the number of sonication runs done for each tube containing *C. elegans* pellets until the solution appears cloudy and translucent carcasses are visible.

3) Total protein may have been lost in the desalting spin-column, but can be recovered by repeating **step G5**.

4) Use a positive control for spectrophotometer readings: In the case that absorbance measurements do not increase as expected with increasing protein concentration in BSA standards, the BCA may have been set up incorrectly and/or the kit may require replacement.

**Problem 3:** The TurboID enzyme does not sufficiently label proteins when assessed by western blotting (i.e., lanes from TurboID animals present a biotinylation signal that resembles your TurboID-negative control).

Possible causes:

1) The TurboID construct is lowly expressed in the worm. This can be because it is expressed from an extrachromosomal array, so not all worms in a population express the enzyme. It can also be because the enzyme has not been codon-optimized for expression in the worm. Finally, it is possible that the tissue targeted for proximity labeling constitutes a small percentage of the worm’s total body volume.

2) The enzyme itself is compromised (e.g., misfolded) due to additional tags.

3) There is insufficient biotin provided for labeling, or biotin has degraded.

Solutions:

1) Consider integrating the extrachromosomal array to improve TurboID-positive cell representation in total protein lysates. This is critical when the enzyme has very low expression, i.e., in limited numbers of cells. Codon optimization may also improve expression levels.

2) Perform a fractionation experiment to isolate insoluble (misfolded) vs. soluble (folded) fractions from total protein. Check these fractions for enzyme expression by western blot.

3) Look up the expiration date for your biotin powder reagent. Assuming that the powder is within date, remake 1 M biotin stock solutions fresh before supplementing them into *E. coli* MG1655 *BioB::kan*. Refer to the protocol by Sanchez et al. for further suggestions (see “Problem 4” in [26]).

**Problem 4:** Unsuccessful attempt to enrich biotinylated proteins by streptavidin-mediated pull-down, i.e., no biotinylated proteins visible during a western blot (for samples from **section G**).

Possible cause: The sample is too diluted, such that RIPA constitutes the majority of the sample volume, rather than TBST, and its components may be impacting the capacity for streptavidin to bind to biotinylated proteins.

Solution:

1) Perform a buffer exchange with the supernatant from the streptavidin-mediated pull-down using desalting spin columns and TBS-P (TBST, but with 1× final concentration of protease inhibitor and no Tween-20). For subsequent biological replicates, use TBS-P instead of RIPA for **section G**.

2) Refer to the protocol by Sanchez et al. for further guidance (see “Problem 8” in [26]).

**Problem 5:** Peptide recovery is low.

Possible causes:

1) DTT, iodoacetamide, or trypsin reagents may have expired.

2) There may be too much background carried over from your pull-down.

Solutions:

1) Check expiry dates for reagents to ensure they are viable. Retry the experiment with freshly made DTT, iodoacetamide, and trypsin solutions.

2) Employ more stringent washing protocols for streptavidin-conjugated magnetic beads after enriching for biotinylated proteins (see “Problem 9” in [26] for more information).

**Problem 6:** There are very few protein identities provided by the MASCOT search of the LC–MS/MS data (n < 100).

Possible cause: This is likely due to an issue with the LC–MS/MS run, or issues with TurboID labeling (Problem 3 above).

Solution: For LC–MS/MS issues, we suggest referring to the user manuals for the mass spectrometer used to troubleshoot this issue. A detailed troubleshooting guide is provided in [53].

**Problem 7:** The peptide summary format available on MASCOT cannot be read by the custom Python code.

Possible cause: You are using a newer version of MASCOT that does not have the peptide summary report option readily available.

Solution: Delete “_2” from the webpage URL of the search results from MASCOT. For example, change the URL http://localhost/mascot/cgi/master_results_2.pl?file=..%2Fdata%2FF981123.dat to http://localhost/mascot/cgi/master_results.pl?file=..%2Fdata%2FF981123.dat. This will change the format to a peptide summary report. Note that newer MASCOT versions likely will not support this solution.

**Problem 8:** Software from this protocol does not work as described in this protocol.

Possible cause: You are using a different software version that is incompatible with the data.

Solution: Download software with the same version as in this publication. As we have not explicitly tested other software versions, we cannot conclude if different versions will function in the same way as described here.
